# Electrosynthesis of ammonia with high selectivity and high rates via engineering of the solid-electrolyte interphase

**DOI:** 10.1016/j.joule.2022.07.009

**Published:** 2022-09-21

**Authors:** Shaofeng Li, Yuanyuan Zhou, Katja Li, Mattia Saccoccio, Rokas Sažinas, Suzanne Z. Andersen, Jakob B. Pedersen, Xianbiao Fu, Vahid Shadravan, Debasish Chakraborty, Jakob Kibsgaard, Peter C.K. Vesborg, Jens K. Nørskov, Ib Chorkendorff

**Affiliations:** 1 Department of Physics, Technical University of Denmark, Kongens Lyngby, Denmark

**Keywords:** electrochemical ammonia synthesis, lithium-mediated nitrogen reduction, high selectivity, high rates, solid-electrolyte interphase

## Abstract

Ammonia is a large-scale commodity essential to fertilizer production, but the Haber-Bosch process leads to massive emissions of carbon dioxide. Electrochemical ammonia synthesis is an attractive alternative pathway, but the process is still limited by low ammonia production rate and faradaic efficiency. Herein, guided by our theoretical model, we present a highly efficient lithium-mediated process enabled by using different lithium salts, leading to the formation of a uniform solid-electrolyte interphase (SEI) layer on a porous copper electrode. The uniform lithium-fluoride-enriched SEI layer provides an ammonia production rate of 2.5 ± 0.1 μmol s^−1^ cm_geo_^−2^ at a current density of −1 A cm_geo_^−2^ with 71% ± 3% faradaic efficiency under 20 bar nitrogen. Experimental X-ray analysis reveals that the lithium tetrafluoroborate electrolyte induces the formation of a compact and uniform SEI layer, which facilitates homogeneous lithium plating, suppresses the undesired hydrogen evolution as well as electrolyte decomposition, and enhances the nitrogen reduction.

## Highlights

• LiF-enriched SEI layer is beneficial to achieve high selectivity
• LiBF_4_ electrolyte induces the formation of a compact and uniform LiF-enriched SEI layer
• Porous Cu electrode combined with LiBF_4_ realizes both high selectivity and high rates
• NH_3_ distribution in different phases is highly dependent on the lithium salts used

## Context & scale

Lithium-mediated nitrogen reduction (LiNR) is a promising pathway to produce ammonia (NH_3_) in a modular and distributed manner, but the process is still limited by low NH_3_ production rate and selectivity. Herein, we establish a theory modeling approach to screen lithium salts for the LiNR process and find that fluorine-based electrolytes are beneficial to achieve a high selectivity due to the presence of lithium fluoride (LiF) in the solid-electrolyte interphase (SEI) layers. We demonstrate that a record selectivity and NH_3_ production rate at a current density of −1.0 A cm_geo_^−2^ under 20 bar N_2_ are achieved by using lithium tetrafluoroborate electrolyte in combination with a highly porous copper electrode. Experimental analysis uncovers that a compact and uniform LiF-enriched SEI layer facilitates even lithium deposition and suppresses the uncontrolled electrolyte degradation. These findings provide new insights into the development of an industrially feasible route for electrochemical NH_3_ synthesis.

## Graphical abstract

**Figure:**
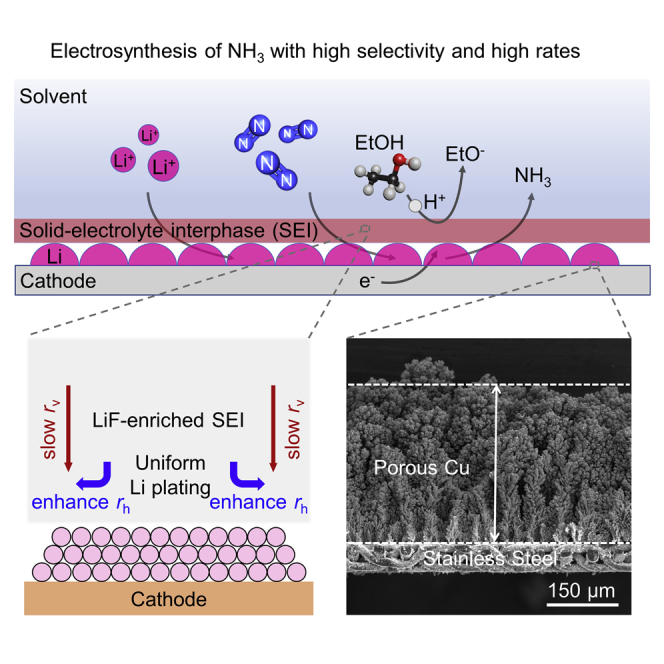

## Teaser

Achieving high selectivity at a commercially relevant current density holds the key to the practical applications of electrochemical NH_3_ synthesis. Here, a record selectivity and NH_3_ production rate at a current density of −1.0 A cm_geo_^−2^ under 20 bar N_2_ are achieved through a combination of highly porous Cu electrode and LiBF_4_-based electrolyte. Theoretical calculations and experimental analysis suggest that a compact and uniform LiF-enriched SEI layer facilitates even lithium plating and suppresses the undesired electrolyte decomposition.

## Introduction

Ammonia (NH_3_) is one of the most abundantly produced chemicals, with an annual production exceeding 182 million tonnes.^1^ Around 80% of the synthesized NH_3_ is used in the fertilizer industry, but it is also regarded a promising carbon-free energy carrier to replace fossil fuels.^2^^,^^3^ Currently, the Haber-Bosch process is the predominant pathway to produce NH_3_ by passing N_2_ and H_2_ over an iron-based catalyst at high temperatures (350°C–450°C) and high pressures (150–200 bar).^4^^,^^5^ The process consumes more than 1% of the global energy supply and leads to about 1.3% of the global CO_2_ emission,^6^^,^^7^ mainly associated with the production of H_2_ from hydrocarbon feedstocks. In addition, considering the extreme operating conditions and the required on-site hydrogen production, this process requires large industrial plants, which are capital intensive. Alternatively, electrochemical NH_3_ synthesis in principle provides a simple route that can be based on renewably generated electricity, which will reduce the CO_2_ footprint, and is also compatible with small-scale facilities that produce NH_3_ in a modular and distributed manner.

**Figure 1:**
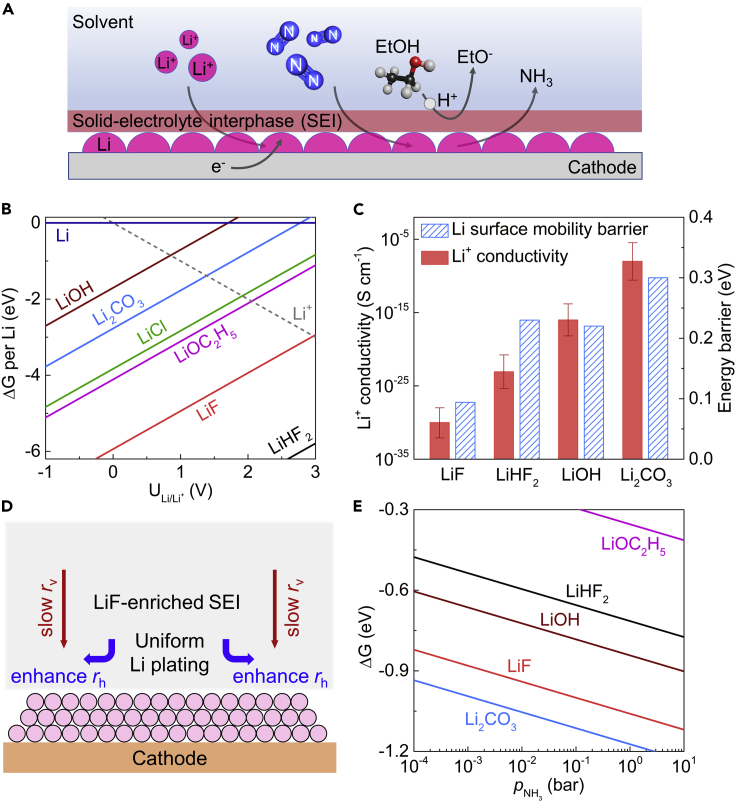
Theoretical investigation of the SEI layer by using different lithium salts (A) Schematic of the mechanism for Li-mediated ammonia synthesis. Although the accurate mechanisms are still not entirely understood, it is broadly believed that this LiNR process relies on the metallic lithium reduced from Li^+^ to dissociate N_2_ followed by a sequence of electron and proton transfers to form NH_3_ with suitable proton donors. (B) Calculated Gibbs formation free energy of Li-containing compounds as a function of voltage (versus Li/Li^+^). (C) The Li^+^ conductivity and energy barrier of Li surface mobility for the Li_2_CO_3_, LiOH, LiHF_2_, and LiF at the operating voltage (U_Li/Li+_ = 0 V). The error bars represent the uncertainty of calculated Li^+^ conductivity. (D) Schematic illustration of proposed Li diffusion model for a LiF-enriched SEI layer during the LiNR process. (E) The Gibbs adsorption free energy of NH_3_ on different Li-containing compounds.

Currently, the only known reliable method of electrochemical NH_3_ synthesis at ambient temperature is lithium-mediated nitrogen reduction (LiNR), which was first reported by Fichter et al. in 1930^8^ and later studied by Tsuneto et al. in the 1990s.^9^^,^^10^ There have been many claims of synthesizing NH_3_ from N_2_ electrochemically in this field, but most of those reports were highly questionable, due to a lack of scientific rigor necessary to prove that the NH_3_ originated from activated N_2_.^11^^,^^12^ Solid evidence with a method for validating that the N_2_ is activated for this process was first provided by our group by using a proper gas cleaning procedure and ^14^N_2_ and ^15^N_2_ isotopes.^11^ Although the accurate mechanisms are still not entirely understood, it is broadly believed that this LiNR process relies on the metallic lithium reduced from Li^+^ to dissociate N_2_ followed by a sequence of electron and proton transfers to form NH_3_ with suitable proton donors or so-called sources (Figure 1A).^13^^,^^14^ The LiNR process was revisited by several groups recently,^11^^,^^13^, ^14^, ^15^, ^16^, ^17^, ^18^, ^19^, ^20^ and the typical reported faradaic efficiency (FE) is around 5%–20% at ambient conditions with NH_3_ production rate less than 0.01 μmol s^−1^ cm_geo_^−2^.^11^^,^^14^^,^^16^^,^^17^ Recently, Suryanto et al. has reported 69% FE at a current density of −0.022 A cm_geo_^−2^ and an NH_3_ production rate of 0.053 μmol s^−1^ cm_geo_^−2^ by using phosphonium salt as a proton carrier under 20 bar pressure.^18^ Our recent work has demonstrated 78% FE at a current density of −0.004 A cm_geo_^−2^ achieved by adding 0.6 to 0.8 mol % oxygen to the 20 bar N_2_ atmosphere, which is attributed to the modification of the solid-electrolyte interphase (SEI) layer formed between the active (lithium) surface and the electrolyte during operation.^21^

The SEI layer over the electrode surface is mainly composed of electrolyte decomposition products, including various inorganic and organic components, known in the lithium-ion battery field to be ion conducting but electron insulating.^22^^,^^23^ This passivation layer is likely a crucial player in determining the stability and performance of the LiNR process. First, it may help improve the system’s stability by avoiding excess electrolyte decomposition and lithium dendrite formation. Second, the SEI layer is a vital factor in determining the relative diffusion rates of Li^+^, H^+^, and N_2_ (*r*_Li_, *r*_H_, *r*_N2_), which are the critical variables determining the rate and selectivity (Figure 1A).^13^^,^^21^ However, the exact composition and functionality of the SEI layer in the LiNR process remains largely unexplored. Further, our group has found that modifying the SEI layer by the addition of oxygen in the N_2_ feed can dramatically increase the FE up to 78%.^21^ To overcome the gap of low current density, we also proposed increasing the current density (e.g., −0.1 A cm_geo_^−2^) by the use of high surface area copper electrodes.^24^ The challenge still remains to achieve high FE and a commercially relevant current density (i.e., around −1.0 A cm_geo_^−2^) in a single system.

This is the challenge we address in this work by modifying the SEI layer and improving the accessible surface area (per geometric surface area) simultaneously. We concentrate on determining the dynamic changes of the SEI under different experimental conditions and understanding how the SEI layer improves the performance of the LiNR process by tuning the ionic conductivity. We study several electrolytes and suggest that a fluorine-based electrolyte is the best. In combination with a highly porous Cu electrode, we demonstrate 95% ± 3% FE at a current density of −0.1 A cm_geo_^−2^ under 20 bar N_2_. In addition, we show a FE of 71% ± 3% is achievable at a current density of −1.0 A cm_geo_^−2^, delivering an NH_3_ production rate of 2.5 ± 0.1 μmol s^−1^ cm_geo_^−2^. The new results are attributed to the uniform lithium fluoride (LiF)-enriched SEI layer that facilitates even lithium deposition and suppresses the uncontrolled electrolyte degradation. The different SEI layers were characterized with X-ray photoelectron spectroscopy (XPS) and X-ray diffraction (XRD), where we confirmed the presence of LiF. Moreover, we also found that the produced NH_3_ does not only exist in the electrolyte but also in the gas phase and in the deposited layer with SEI, and the NH_3_ concentration distribution in different phases is highly related to the lithium salt used.

## Results

### Theoretical investigation of the SEI layer

We first present an analysis of the thermodynamic stability of different Li-containing compounds that form in the SEI layer. In the following discussion, we only focus on the most stable compounds that are not soluble in tetrahydrofuran (THF), since these are likely to be the main components in the SEI, thereby determining the intrinsic properties. Other phases could also be part of the SEI, but most likely not in large quantities compared with the most stable materials. Figure 1B shows the free energy (Pourbaix diagram) versus potential of the 6 most stable phases that could form in the SEI layer at different potentials based on experimental stability data.^25^ The full Pourbaix diagram including all possible experimentally reported Li-containing compounds is shown in Figure S1. The two most stable phases using LiClO_4_ as the lithium salt and pure N_2_ gas are LiOC_2_H_5_ and Li_2_CO_3_, since LiCl is soluble in THF.^26^ This is in agreement with the literature for Li-ion batteries,^27^ where it is found that organic salts (e.g., LiOC_2_H_5_) near the SEI/electrolyte interface are porous, so that Li^+^ can be transported with other anions through this organic outer layer of the SEI, whereas a dense inorganic layer (e.g., Li_2_CO_3_) blocks further anion diffusion. Therefore, the transport mechanism of Li^+^ in the inorganic layer is most likely based on Li defect formation and defect diffusion. As shown in Figure 1B, when using LiBF_4_ instead of LiClO_4_ as the lithium salt in the electrolyte, the two most stable solid phases in the SEI are LiHF_2_ and LiF. Although Li_2_B_6_O_10_, Li_2_B_4_O_7_, and NH_3_·BF_3_ are even more stable than LiHF_2_ and LiF (Figure S1), they are more soluble in THF.^28^

The elementary steps in the LiNR include the diffusion of Li^+^, H^+^, and N_2_ species from bulk electrolyte through SEI toward the electrode surface followed by Li deposition, H_2_ formation, and NH_3_ formation. Due to the presence of the SEI, the diffusion of these three species is rather slower than that of H_2_ and NH_3_ formation at extreme operating potential (∼−3 V).^13^^,^^21^ Therefore, the diffusion rates of Li^+^, H^+^, and N_2_ species in the SEI are the overall rate-limiting steps in the LiNR.^13^^,^^21^ For Li^+^ transport properties, we conducted a comprehensive first-principles study of possible Li point-defect formation energies in Li_2_CO_3_, LiOH, LiHF_2_, and LiF (Figure S2) and identify the dominating defects at the applied voltage range. The Li^+^ conductivity is related to the defect concentration and the diffusivity via the Nernst-Einstein equation.^29^ Based on random-walk theory,^29^ the diffusivity is determined by the migration barrier, which we calculate by the climbing image nudged elastic band (CI-NEB) method,^30^ whereas defect concentration depends on the defect formation energy (see experimental procedures). As shown in Figure 1C, at the operating voltage (0 V versus Li/Li^+^), the calculated Li^+^ conductivity in LiHF_2_ and LiF is several orders of magnitudes lower than that of Li_2_CO_3_. The presence of LiF results in a decrease of *r*_Li_ relative to Li_2_CO_3_. The diffusion rates of proton and N_2_ are estimated via Fick’s first law. Consider the case of linear (one-dimension) diffusion of proton from bulk electrolyte through SEI approaching the electrode surface, the flux of proton $J_{H^{+}}\left(x,t\right)$ at given position x at a time t is proportional to the concentration gradient $C_{H^{+}}$, that is, $J_{H^{+}}\left(x,\;t\right)=D_{H^{+}}\frac{\partial C_{H^{+}}\left(x,\;t\right)}{\partial x}$. Since the N_2_ reduction and H_2_ evolution reactions are fast enough at the very negative potential (<−3 V), the proton and N_2_ concentration at electrode surface (x=L) is approximated to be zero. Therefore, the diffusion rates of proton and nitrogen are estimated by $D_{H^{+}}=\frac{3J_{NH_{3}}}{FE}\frac{L}{C_{H^{+}}\left(0,\;t\right)}$ and $D_{N_{2}}=J_{NH_{3}}\frac{L}{C_{H^{+}}\left(0,\;t\right)}$, where L is the thickness of the SEI chosen to be 10–100 nm.^31^ As shown in Table S1, there is a small change of *r*_H_ and *r*_N2_ at different experimental conditions (i.e., different main components of the formed SEI) relative to *r*_Li_. Therefore, the decreased *r*_Li_ caused by the presence of LiF should lead to a considerable FE increase according to the microkinetic modeling reported in our previous work.^13^^,^^21^ The reason is that fewer electrons are “wasted” depositing Li relative to electrons used in reducing nitrogen. A similar phenomenon is also observed in our previous work, where by adding small amounts of oxygen,^21^ the formation of LiOH competes with that of Li_2_CO_3_ in the SEI using a LiClO_4_ salt (Figure S3), leading to a FE increase from 25% to 78%. The resulting FE due to the changes of Li diffusion rates in different SEI components are summarized in Figure S4.

In the beginning, the Li^+^ transporting through bulk electrolyte is reduced to Li metal immediately and deposited on the electrode surface. The active Li metal will spontaneously decompose electrolyte, resulting in the growth of SEI. An ideal SEI is electron insulating to prevent continuous electrolyte decomposition, yet ion conducting to lithium ions. In the presence of the SEI, the Li^+^ that go through the SEI is deposited as metallic Li on the electrode surface. The deposition of metallic Li results in the formation of the Li dendrite if the two-dimensional Li mobility parallel to the SEI and the electrode surface is rather low, leading to a poor homogeneity of the SEI.^32^ Here, we investigated the Li mobility in the surfaces of Li_2_CO_3_, LiOH, LiHF_2_, and LiF. The most probable/stable surfaces for each species are selected based on the surface phase diagram (see Figures S5–S8). As shown in Figure 1C, LiF exhibits a 0.09 eV migration barrier for Li surface mobility, which is lower than that of LiOH (0.22 eV) and Li_2_CO_3_ (0.3 eV). Therefore, the LiF-enriched SEI could improve an uneven electrodeposition of lithium by enhancing Li surface mobility, thus a more homogeneous SEI, and the similar phenomenon is also observed in Li-ion batteries.^33^ Furthermore, LiF is more electrically insulating and has a wider electrochemical stability window than Li_2_CO_3_ (see Figure S9), thus creating a better passivated electrode surface to prevent undesired side reactions between deposited lithium and electrolyte.

Figure 1D is a schematic illustration of our proposed Li^+^ diffusion model for a LiF-enriched SEI layer during the LiNR process. The *r*_Li_ consists of diffusion rates in two directions, i.e., vertical and horizontal, and denoted as *r*_v_ (Li^+^ diffusion rate through the SEI, v: vertical) and *r*_h_ (Li^+^ diffusion rate on the surface, h: horizontal), respectively. The model suggests that by decreasing *r*_v_ while enhancing *r*_h_, the LiF-enriched SEI layer enables a homogeneous Li^+^ flux and suppresses Li dendrite formation, thus leading to a further increase in FE. In addition, as shown in Figure 1E, we find that NH_3_ molecules can easily be absorbed in the bulk and the surface of different Li-containing phases, e.g., LiOC_2_H_5_, Li_2_CO_3_, LiOH, LiHF_2_, and LiF, which suggests that the produced NH_3_ could be possibly trapped in the SEI layer. It also should be noted that the NH_3_ formed using LiBF_4_ as lithium salt can be trapped as NH_3_·BF_3_, which is easily soluble in THF and ethanol. As shown in Figure S1, the Gibbs formation free energy of Li_x_H_y_N_z_ (Li_3_N, Li_2_NH, and LiNH_2_) species per Li atom is several eV (at least 6 eV at the operation voltage) higher than that of LiHF_2_, LiF, and NH_3_·BF_3_, so the portion of Li_x_H_y_N_z_ in the SEI is much less than that of LiHF_2_, LiF, and NH_3_·BF_3_. It indicates that the produced NH_3_ may mainly exist in the electrolyte rather than in the SEI layer by using LiBF_4_ as lithium salt.

### Experimental demonstration

**Figure 2:**
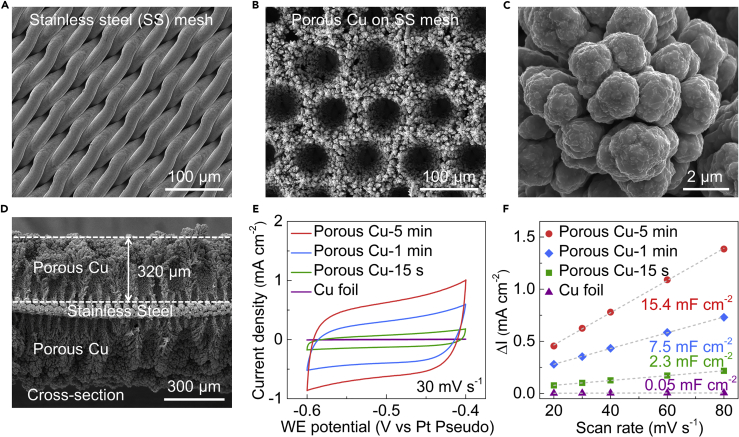
Fabrication of porous Cu electrodes for Li-mediated ammonia synthesis (A–C) Representative SEM images of the stainless steel (SS) mesh (A) and porous Cu electrode (B and C). (D) Cross-section SEM images of the porous Cu electrode. (E) Cyclic voltammetry of different porous Cu electrodes at scan rate of 30 mV s^−1^. (F) Current density change versus scan rate of different porous Cu electrodes and the calculated specific capacitances. The change in current density was determined at −0.5 V versus reference electrode.

Motivated by theoretical results, we choose two typical fluorine-based lithium salts, i.e., LiBF_4_,^14^^,^^15^^,^^18^^,^^20^ LiPF_6_, and the widely used LiClO_4_,^9^^,^^10^^,^^16^^,^^21^ as the model systems. It should be noted that Lazouski et al. have first reported 18% FE by using LiBF_4_ under ambient pressure,^14^ and then, LiBF_4_ was also used by different groups in this field.^18^^,^^20^ However, the comprehensive investigations on the effect of different lithium salts on the SEI layer are unexplored. In contrast to our previous study, a stainless steel (SS) mesh was used as substrate rather than a Ni foam,^15^ in order to allow the controlled growth of porous Cu using the hydrogen bubble template method.^24^ The geometrical surface area was defined as the front of a 0.5 × 0.4 cm^2^ SS mesh or Cu foil. The detailed procedures of Cu deposition on the SS mesh are shown in the experimental procedures. The Cu was chosen as the electrode material here because Cu has difficulties alloying with lithium electrochemically in organic electrolyte.^9^^,^^10^^,^^34^ Scanning electron microscopy (SEM) images show that highly structured Cu with well-ordered pores self-assemble on the SS mesh (Figures 2B–2D). The high-resolution SEM image in Figure 2C shows that the highly structured Cu consists of connected Cu particles with a diameter of ∼1 to 3 μm. As shown in Figures 2D and S10A, the thickness of the deposited Cu can be well controlled and tuned from 110 to 470 μm by changing the deposition time. It should be noted that changing other deposition parameters, e.g., applied current (Figure S11), could also change the deposition thickness and the porous structure. We also would like to point out that the porous electrode can also be made by other transition metals using the hydrogen bubble template method, such as Ni, Co, etc.,^35^, ^36^, ^37^, ^38^ which could potentially also used for LiNR process. To determine the electrochemical surface area (ECSA) of the porous Cu synthesized with different deposition time, i.e., 15 s, 1 min, and 5 min (denoted as porous Cu-15 s, porous Cu-1 min, and porous Cu-5 min), capacitive cycling was employed to measure the specific capacitances. The cycling voltammetry (CV) curves of the as-made porous electrodes and the Cu foil at various scan rates are shown in Figures 2E and S12. It can be seen that current density and the average area of the porous Cu electrodes are much higher than the Cu foil, implying a much higher specific capacitance and ECSA. As shown in Figure 2F, the porous Cu electrodes displays much higher current densities at the same scan rates compared with the Cu foil. The calculated specific capacitance of the porous Cu-5 min is 15.4 mF cm_geo_^−2^, which is 300 times higher than the Cu foil (0.05 mF cm_geo_^−2^). Therefore, the ECSA of 308 cm^2^ was determined for the porous Cu-5 min electrode with geometric area of 1 cm^2^, and such a considerable increase of ECSA is attributed to the deposited porous Cu with high surface area.

**Figure 3:**
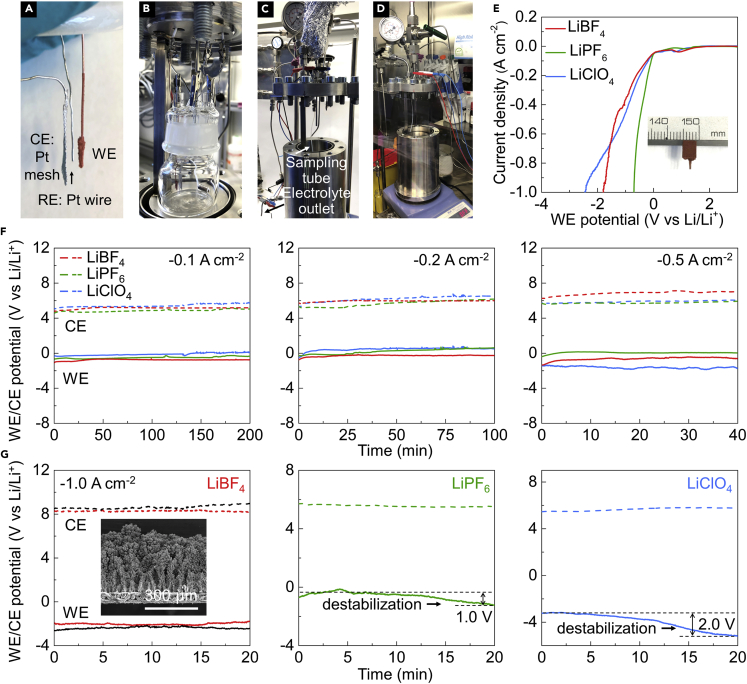
Electrochemical performance of Li-mediated ammonia synthesis (A and B) Digital photos of the setup for working, counter, and reference electrodes (A) and the glass cell sitting in the autoclave (B). The distance between WE and RE was fixed around 0.5 cm for all the experiments. (C and D) Digital photos of the autoclave sitting in the fume hood (C) and Ar glovebox (D). (E) LSV of the porous Cu electrode using different lithium salts. Inset in (E) is a digital photo of the porous Cu electrode (0.2 cm_geo_^2^). (F and G) Chronopotentiometry (CP) of the porous Cu electrode at current densities of −0.1, −0.2, and −0.5 A cm_geo_^−2^ (F) and −1.0 A cm_geo_^−2^ (G) with different lithium salts. Inset in (G) is the cross-section SEM image of the porous Cu electrode without porous Cu on the backside. The black lines represent the data of the porous Cu electrode without porous Cu on the backside. All the experiments here were using the porous Cu electrodes that were synthesized at the same condition, and 2 M lithium salt in tetrahydrofuran solutions containing 1 vol % ethanol under 20 bar N_2_. A total charge of 240 C was passed for the CP measurements at varied current densities.

In order to investigate the current density achievable, porous Cu-15 s, porous Cu-1 min, and porous Cu-5 min were selected for the standard test using the widely used LiClO_4_ electrolyte. As shown in Figures 3A–3D, all the experiments were carried out in a custom-designed autoclave with a glass cell containing 30 mL electrolyte under 20 bar N_2_. The electrolyte was 2 M lithium salt in THF containing 1 vol % ethanol (0.17 M ethanol). The N_2_ used in the experiments was 99.9999% pure and additionally cleaned with purifiers (NuPure) to reduce the nitrogen-containing impurities to parts per trillion levels. The as-prepared porous Cu electrode (0.2 cm_geo_^2^), Pt mesh (1 cm_geo_^2^), and Pt wire were used as working electrode (WE), counter electrode (CE), and pseudo-reference electrode (RE), respectively (Figure 3A). As shown in the linear sweep voltammetry (LSV, Figure S13), current densities of -0.1, -0.3, and -1.0 A cm_geo_^−2^ were achieved by using the porous Cu-15 s, porous Cu-1 min, and porous Cu-5 min, respectively. Therefore, systematic experiments with different lithium salts were further conducted using the porous Cu-5 min electrode that achieved a current density of −1.0 A cm_geo_^−2^. As shown in Figure 3E, the current density of −1.0 A cm_geo_^−2^ can also be reached by using the fluorine-based electrolyte, i.e., LiBF_4_ and LiPF_6_. Potentiostatic electrochemical impedance spectroscopy (PEIS) was employed to measure the bulk electrolyte resistance (Figure S14). The electrolyte resistance for the three electrolyte formulations in descending order is thus as follows: LiBF_4_ > LiClO_4_ > LiPF_6_.

Constant-current densities from −0.1 to −1.0 A cm_geo_^−2^ were applied for the chronopotentiometry (CP) measurements when using different lithium salts, until the total passed charge reached 240 C. As shown in Figure 3F, the WE and CE potentials are quite stable for all the lithium salts at various constant-current densities from −0.1 to −0.5 A cm_geo_^−2^ within the investigated time period. In contrast, the WE potential of the porous Cu electrode using LiPF_6_ and LiClO_4_ exhibits destabilization during CP measurement at a current density of −1.0 A cm_geo_^−2^, although the WE potential using LiBF_4_ is stable in that period (Figure 3G). Specifically, the WE potential shows a drop of ∼1.0 and 2.0 V for the porous Cu electrode using LiPF_6_ and LiClO_4_, respectively, which is indicative of unstable SEI layers and severe electrolyte decomposition. We have here chosen to define the geometric area as only one side of the SS mesh. Nevertheless, one should be aware that the porous Cu was deposited on both sides as seen in Figure 2D. In order to investigate the influence of the porous Cu deposit on the backside of the electrode, we removed the Cu deposit on the backside (inset in Figures 3G and S10B) and tested this electrode again at current density of 1 A cm_geo_^−2^. It also shows the similar stability (black lines in Figure 3G), which indicates that the backside does not significantly influence the electrochemical stability. After CP measurement at a current density of −1.0 A cm_geo_^−2^, the similar morphology as the pristine electrode is shown for the porous Cu electrode using LiBF_4_ (Figure S15), indicative of a stable porous structure during the electrochemical tests. In addition, as shown in Figure S16, the calculated specific capacitance of the porous Cu electrode using LiBF_4_ after the CP measurements is 15.2 mF cm_geo_^−2^, which is also similar to the pristine electrode (15.4 mF cm_geo_^−2^).

As shown in the Figure S17, both LiPF_6_ and LiClO_4_ electrolytes turn black after CP measurements at −1.0 A cm_geo_^−2^, whereas the LiBF_4_ electrolyte only shows a mild color change. In addition, the black electrolyte became highly viscous within a few hours post-electrochemistry (Figure S18), which is ascribed to the serious electrolyte decomposition (Figure S19), specifically THF oxidation that might lead to production of polymers.^39^ The changes of the electrolyte color at varied constant-current densities from −0.1 to −1.0 A cm_geo_^−2^ are shown in Figure S20. This shows the general instability of the electrolyte under these experimental conditions, which is particularly prominent for the LiPF_6_ and LiClO_4_ salts. Moreover, it is obviously seen that the electrolyte color of LiClO_4_ darkens as the current density increases, which can be attributed to the high CE potential at high current density that might lead to more THF oxidation reactions. However, the LiBF_4_ only show a mild color change after CP measurements even at −1.0 A cm_geo_^−2^. We believe that lowering the CE potential below THF oxidation by utilizing hydrogen oxidation reaction (HOR) at the CE will help to solve this issue, which is desirable for the follow-up study. Another difference seen in the images is the huge variances of the deposited layers over the porous Cu electrode using different lithium salts (Figure S17). The deposited layer using LiBF_4_ looks much thinner than that of LiPF_6_ and LiClO_4_, which indicates a compact SEI layer without excess organic components. We also point out that the deposited layers shown in this work is without damages from degassing and air exposure. This is an advantage of the modified autoclave placed inside an Ar glovebox (Figure 3D), which enables separation of the electrode from the electrolyte prior to depressurization, such that the SEI remains intact. This is important, as the depressurization from 20 bar and subsequent air exposure destroys the structure and composition of the SEI layer, which precludes the following XPS investigations on the different SEI layers and deposits.

### Efficiency of the Li-mediated ammonia synthesis

**Figure 4:**
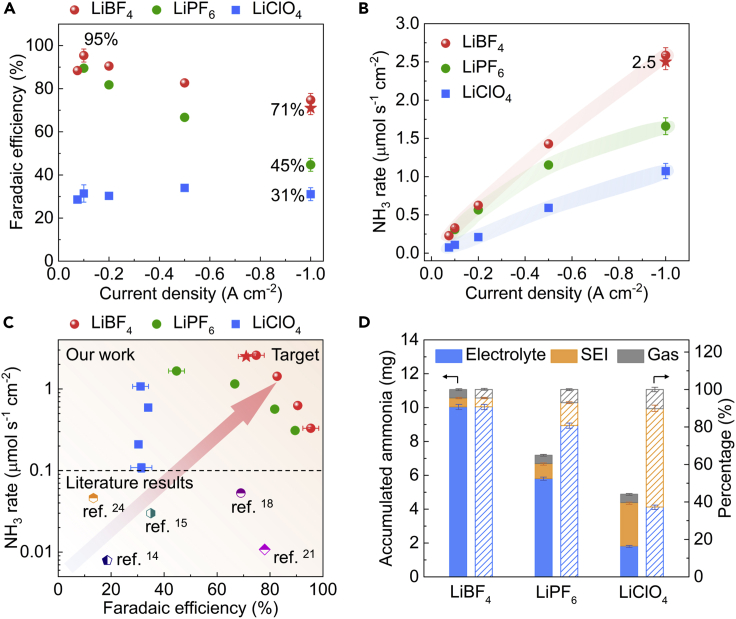
Efficiency of the Li-mediated ammonia synthesis (A and B) Faradaic efficiencies (A) and NH_3_ production rates (B) of the porous Cu electrode using different lithium salts at current densities ranging from −0.075 to −1.0 A cm_geo_^−2^. The shadows in (B) are guides to the eye. (C) A comparison of NH_3_ production metrics at ambient temperature between our work and reported highest rates in non-aqueous electrolytes in the literature. (D) Accumulated NH_3_ in the electrolyte, deposited layer including SEI, and gas phases using different lithium salts at −1.0 A cm_geo_^−2^. The left and right y axis in (D) represents the weight and corresponding percentage of NH_3_, respectively. The calculated faradaic efficiencies and NH_3_ production rates at current densities ranging from −0.1 to −1.0 A cm^−2^ are based on the experiments shown in Figure 3. The error bars represent the standard deviation of independent repeats of the same experiment (n ≥ 3).

The FE was determined at the end of the experiment, where the accumulated NH_3_ was detected in the electrolyte solutions by a modified indophenol blue method.^11^ More details can be found in the experimental procedures. Figure 4A shows the FE of the porous Cu electrode using different lithium salts for different CP measurements with current densities ranging from −0.075 to −1.0 A cm_geo_^−2^. The porous Cu electrode with LiBF_4_ salt exhibits a remarkable 95% ± 3% FE at a current density of −0.1 A cm_geo_^−2^. Furthermore, it is striking that a relatively high FE of 75% ± 3% is achieved at a current density of −1.0 A cm_geo_^−2^, which is far higher than that using LiPF_6_ (45% ± 3% FE) and LiClO_4_ (31% ± 3% FE) salts. The porous Cu electrode without porous Cu on the backside also shows a similar FE (71% ± 3%, star in Figure 4A) to the porous Cu electrode with Cu on the two sides (75% ± 3%, sphere in Figure 4A) by using LiBF_4_ salt, which indicates that the backside does not significantly influence the FE. The FE drops rapidly at high current densities for the LiPF_6_ salt, i.e., from 90% FE at −0.1 A cm_geo_^−2^ to the aforementioned 45% ± 3% FE at −1.0 A cm_geo_^−2^, whereas that using LiClO_4_ salt shows a relative stable FE around 31% ± 3% at varied current densities. The rapid FE drop when using LiPF_6_ salt at high current densities is attributed to the severe electrolyte decomposition and unstable SEI layers, considering the poor thermal stability of LiPF_6_ and the potentially produced joule heat at high current densities. It has been widely investigated and proven within the Li-ion battery field that LiPF_6_-based electrolyte has poor stability at elevated temperatures, e.g., 60°C, and the SEI layer is unstable at elevated temperature, especially in the presence of LiPF_6_.^40^, ^41^, ^42^, ^43^ In addition, we also ran the CP measurements using Cu foil (1.8 cm^2^) with different salts at −4 mA cm^−2^ until the total passed charge reached 50 C (Figure S21). The standard Cu foil electrode with LiBF_4_ salt exhibits 65% FE, which is also higher than that LiClO_4_, which only achieved 20% FE.

Accordingly, the NH_3_ production rate of the porous Cu electrode using different lithium salts at varied current densities is shown in Figure 4B. The porous Cu electrode using LiBF_4_ exhibits an NH_3_ production rate of 2.5 ± 0.1 μmol s^−1^ cm_geo_^−2^ at a current density of −1.0 A cm_geo_^−2^ based on 71% ± 3% FE of the electrode with the backside deposited Cu removed. Therefore, the backside Cu does not significantly affect the electrochemical stability, FE production rate, and NH_3_ production rate. As show in Figure 4C, the FE and NH_3_ production rates when using LiBF_4_ at a current density of −0.1 A cm_geo_^−2^ (95% ± 3% and 0.33 ± 0.01 μmol s^−1^ cm_geo_^−2^) are, to the best of our knowledge, the highest ever reported for the LiNR process.^14^^,^^15^^,^^18^^,^^21^^,^^24^ More importantly, the reported NH_3_ production rate of 2.5 ± 0.1 μmol s^−1^ cm_geo_^−2^ at a current density of −1.0 A cm_geo_^−2^ is more than one order of magnitude higher than all published literature results.^18^ Although the energy efficiency (EE) is currently also a major concern in the LiNR process, considering Li plating requires large negative potentials (−3.04 V versus standard hydrogen electrode). Based on our previous work,^21^ if all overpotentials are minimized and HOR is utilized at the CE, the optimized resulting EE would be 26%, assuming 80% FE. Thus, as pointed out in our previous publications,^13^^,^^21^^,^^24^ the EE reported here is artificial as it does not take into account the sacrificial proton source. We want to point out here that initially in the 1950s the Haber-Bosch process also exhibited low energy efficiencies of 36% and only reached 62% in the 1990s.^44^ It also should be clarified here that NH_3_ production rate per geometric surface area rather than NH_3_ production rate per ECSA is pursued in this work, which is more relevant to the industrial application.

The EE of the porous Cu electrode using LiBF_4_ at a current density of −1.0 A cm_geo_^−2^ is 7.7% (Table S2), which is well beyond the previously reported value of 2.8% for a 6 min experiment,^15^ 1.5% for a 40 min experiment,^14^ and 2.3% in literature.^24^ In addition, regarding to the long-term stability, the single-compartment cell was used in this work with a sacrificial proton source. There are substantial energy losses related to the anode in the single-compartment cell. Ideally, a two-compartment setup, such as flow cell, should be used for better control of the anode reaction, which allow the HOR to use H_2_, instead of consuming a sacrificial proton source. We also believe that further improvements to the electrolyte conductivity in future can also improve the EE, considering the energy losses from the large electrolyte resistance. The in-depth investigation of the long-term stability of the LiNR system by using flow cell is the subject of our ongoing work, whereas the goal of this work is to demonstrate the remarkable NH_3_ production rates and FE at −1.0 A cm_geo_^−2^ that are possible by modifying the SEI layer using and improving the accessible surface area (per geometric surface area). It is also noteworthy that in our previous work, we have developed a potential cycling procedure that greatly extend the lifetime of the LiNR system, and continuous operation for 125 h was demonstrated.^13^

Additionally, inspired by the theoretical modeling regarding the possibility of NH_3_ being trapped in the SEI layer, we further conducted a separation procedure in the glovebox. First, right after the end of the CP measurements conducted at −1.0 A cm_geo_^−2^, we extracted the electrolyte from the glass cell through a polytetrafluoroethylene (PTFE) sampling tube, whereas the system is still pressurized at ∼20 bar in the autoclave (Figure 3C). Next, degas the system although using an acid trap to collect all the NH_3_ in the gas phase, and finally, open the autoclave and remove the WE with the intact deposited layer. For a comparison, as shown in Figure S22, there is no obvious thick deposit shown on the porous Cu electrode after depressurization from 20 bar without separation procedure, and most of the deposit was broken into pieces and floated on the electrolyte due to the degassing with electrolyte. This indicates that without separation procedure to extract the electrolyte first, the deposited layer can be easily damaged by degassing from 20 bar. Such a separation procedure ensures the SEI layer is damaged as little as possible, which is very important for the following SEI investigations. All the experiments toward investigating of the SEI layer were conducted in an autoclave placed inside an Ar glovebox to avoid air exposure, although completely avoiding O_2_ and H_2_O exposure can never be obtained by such methods as typical H_2_O and O_2_ content in Ar glovebox are in the <0.5 and <0.1 ppm range, respectively. More detailed information about the separation procedure can be found in the experimental procedures.

As shown in Figure 4D, it is clearly seen that the produced NH_3_ is not only present in the electrolyte but also in the gas phase, as well as the deposited layer, which is a composite of the SEI layer and excess deposited species. The distribution of the accumulated NH_3_ in the different phases is strongly dependent on the type of lithium salt used. Particularly, ∼50% of the synthesized NH_3_ was trapped in the deposited layer using LiClO_4_, which is much higher than both LiPF_6_ (∼10%) and LiBF_4_ (∼5%). We would like to point out that the synthesized NH_3_ trapped in the deposited layer could also include the intermediate nitrogen species (reduced from N_2_) that can be easily converted to NH_3_ during the collection step using HCl to dissolve the deposited layer (see experimental procedures). The high amount of NH_3_ trapped in the deposited layer when using LiClO_4_ was possibly caused by the massively thick SEI layer and deposit, which is a sign of severe electrolyte decomposition, especially of the organic components.^39^ The smaller amount of NH_3_ trapped in the deposited layer using LiBF_4_ can be possibly ascribed to the high solubility of NH_3_·BF_3_ in THF and ethanol, which is also revealed by the theoretical results (Figure 1E). Most of the produced NH_3_ is distributed in the electrolyte when using LiBF_4_ salt in the single-compartment cell. However, the flow cell-based system with continuous N_2_ gas flow can be used to potentially change the NH_3_ distribution, leading to more of the produced NH_3_ in the gas phase, which would be ideal for convenient collection and further utilization. It is also noteworthy that the total accumulated NH_3_ reported here are all at the milligram level (Figure 4D), rather than the microgram level reported most commonly in the literature. For example, ∼11 mg NH_3_ was synthesized in a single experiment at different current densities by using 30 mL LiBF_4_-based electrolyte with passed charge of 240 C. Considering the LiNR process has been well established by different groups,^11^^,^^14^^,^^15^^,^^18^^,^^21^ we would like to point out that when produced NH_3_ in this milligram range and using the well-described precautions,^11^ it is not necessary to conduct isotope measurements.

### SEI layer investigations

To further elucidate the effect of different lithium salts on the SEI layer, we purposely designed the short electrochemical experiments in the Ar glovebox using LiBF_4_, LiPF_6_, and LiClO_4_ salts. As shown in Figure S23A, we conducted the LSV measurements for the porous Cu electrodes using different lithium salts and stopped the reaction after the working potential passed lithium plating and reached a current density of −0.1 A cm_geo_^−2^. Then, we followed the same procedures as the separation procedure and collected the electrodes with the intact SEI layers. These short electrochemical experiments are supposed to build only a thin SEI layer without severe electrolyte decomposition (Figure S23B), which is more relevant to the theory insights. All the porous Cu electrodes with deposited layers were loaded into a home-built transfer arm inside an Ar glovebox and evacuated to pressures below 5 × 10^−6^ mbar, followed by a transfer into the XPS chamber with a base pressure below 9 × 10^−10^ mbar (Figure S24). Depth-profiling XPS with different etching times using Ar ions was engaged to probe the elemental composition, chemical state, and depth profile of the SEI layer.

**Figure 5:**
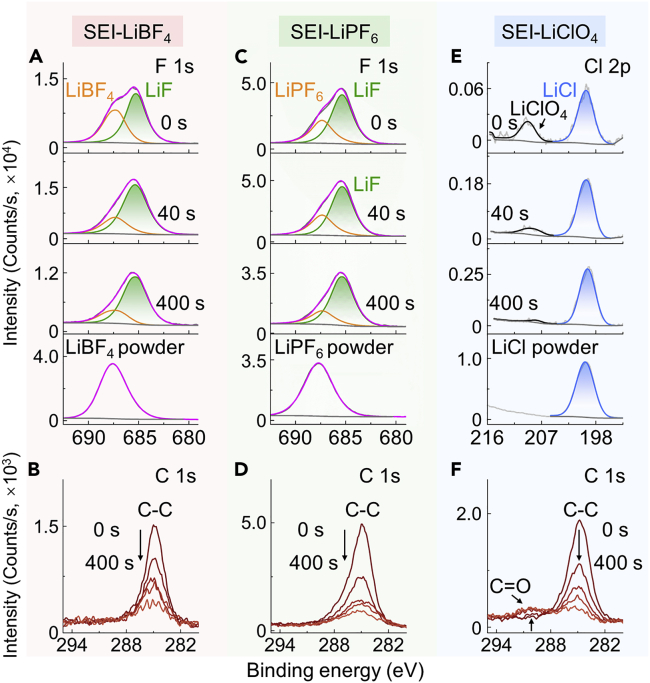
XPS investigation on the SEI layers without degassing damage and air exposure (A and B) Depth-profiling XPS spectra of F 1s (A) and C 1s (B) for the SEI-LiBF_4_. (C and D) Depth-profiling XPS spectra of F 1s (C) and C 1s (D) for the SEI-LiPF_6_. (E and F) Depth-profiling XPS spectra of Cl 2p (E) and C 1s (F) for the SEI-LiClO_4_. The commercial LiBF_4_, LiPF_6_, and LiCl powder were used as reference samples.

As shown in the F 1s spectra of the SEI layer formed using LiBF_4_ (denoted as SEI-LiBF_4_, Figure 5A), the peak centered at 685.5 eV is attributed to LiF,^45^ and the peak at 687.5 eV is well matched with LiBF_4_. Additionally, the ratio of the LiF signal increases as the etching time increases, which indicates a LiF-enriched SEI layer on the porous Cu electrode using LiBF_4_ and confirms the theoretical suggestion. As shown in Figure 5C, similar phenomena were also observed for the SEI layered formed using LiPF_6_ (denoted as SEI-LiPF_6_). Figure 5E shows the Cl 2p spectra of the SEI layer formed using LiClO_4_ (denoted as SEI-LiClO_4_), and the peak centered at 200.0 and 209.5 eV is attributed to LiCl and LiClO_4_, respectively. It is clearly seen that LiCl is derived from the reduced product of LiClO_4_ and is enriched in the SEI-LiClO_4_, which also implies that solvent oxidation might already happened at the beginning of the reaction due to the strongly oxidizing property of LiClO_4_. In addition, as shown in the C 1s spectra (Figures 5B, 5D, and 5F), compared with the SEI-LiBF_4_ and SEI-LiPF_6_, the SEI-LiClO_4_ exhibits a new peak after etching that is well matched with the C=O bond and possibly attributed to the presence of Li_2_CO_3_.^46^

Moreover, the different deposits (shown in Figure S17) on the porous Cu electrodes after CP measurements at a current density of −1.0 A cm_geo_^−2^ were also investigated in the same manner to reveal the possible changes of the deposit after reaction. As shown in Figure S25, the LiF remain enriched in the deposits formed using LiBF_4_ and LiPF_6_ (denoted as Post-LiBF_4_ and Post-LiPF_6_, respectively), and the XRD pattern shown in Figure S26 further confirmed the presence of LiF phase in both Post-LiBF_4_ and Post-LiPF_6_. The signal intensity of the P 2p reduces rapidly compared with that of B 1s during etching (Figures S25B and S25E), indicating the inhomogeneous composition of the Post-LiPF_6_, which could be caused by the serious electrolyte decomposition at high current density due to the poor thermal stability of LiPF_6_. In addition, the C 1s spectra shown in Figures S25H and S27 exhibit that the deposits formed using LiClO_4_ (denoted as Post-LiClO_4_) has a strong peak assigned to the C=O bond, which might indicate more Li_2_CO_3_ accumulated inside. As shown in the N 1s spectra (Figure S28), both Post-LiBF_4_ and Post-LiClO_4_ have a peak centered at 398.6 eV, which is attributed to organic nitrogen species^47^^,^^48^ rather than Li_3_N, as is seen from the Li_3_N reference spectrum. The depth profile of the elemental composition for the different SEI layers clearly shown that F and Li are the predominant elements inside of the Post-LiBF_4_ and Post-LiPF_6_ (Figures S25C, S25F, and S25I), which also indicates the LiF-enriched deposit. However, Li is the main element inside of the Post-LiClO_4_, and more Li is shown inside of the Post-LiPF_6_ compared with Post-LiBF_4_, which could indicate the uncontrolled Li plating using LiPF_6_ and LiClO_4_ at a current density of −1.0 A cm_geo_^−2^. Besides, more C is also shown inside of the Post-1A-LiClO_4_, which further implies the severe electrolyte decomposition at a current density of −1.0 A cm_geo_^−2^.

Therefore, based on the investigations on the different SEI layers formed at the beginning and the deposits formed after CP measurements at a current density of −1.0 A cm_geo_^−2^, the huge FE differences by using LiBF_4_, LiPF_6_, and LiClO_4_ are explained as following: (1) the high FE achieved by using LiBF_4_ and LiPF_6_ at relatively low current densities are ascribed to the LiF-enriched SEI layers that result in a decreased *r*_Li_ with a smaller change of *r*_H_ and *r*_N2_ and promote the LiNR process, which is revealed by the theory modeling results. LiF is beneficial to the uniform lithium plating and builds a better interface to prevent too much undesired side reactions between deposited lithium and the electrolyte. (2) The high FE of 71% ± 3% achieved at a current density of −1.0 A cm_geo_^−2^ by using LiBF_4_ instead of LiPF_6_ and LiClO_4_ is not only attributed to LiF-enriched SEI layer but also due to increased thermal and chemical stabilities of LiBF_4_, which suppresses severe electrolyte decomposition. Particularly, the poor thermal stability of LiPF_6_ and strongly oxidizing property of LiClO_4_ results in a disastrous electrolyte decomposition, which could potentially destroy and complicate the SEI layer formed at the beginning and result in the uncontrolled lithium plating.

## Discussion

In summary, we utilize a theory modeling approach to screen the lithium salts for the LiNR process and find that fluorine-based electrolytes are beneficial to achieve a high FE due to the presence of LiF in the SEI layer. Through systematic experimental demonstrations, the LiBF_4_-based electrolyte is observed as the most stable and promising electrolyte to realize a highly efficient LiNR process. We demonstrate that combined with a highly porous Cu electrode, record FE of 71% ± 3% and NH_3_ production rate of 2.5 ± 0.1 μmol s^−1^ cm_geo_^−2^ at a current density of -1.0 A cm_geo_^−2^ under 20 bar N_2_ are achieved using LiBF_4_-based electrolyte. The good LiNR performance can be explained by the formation of a compact and uniform LiF-enriched SEI layer and the better thermal and chemical stability of LiBF_4_, which enables even lithium plating and suppresses uncontrolled electrolyte degradation. We also find that the NH_3_ distribution in the gas, electrolyte, and deposited layer with SEI is highly dependent on the lithium salts used. We anticipate that our findings offer new insights on developing an industrially feasible pathway for electrochemical NH_3_ synthesis.

## Experimental procedures

### Resource availability

#### Lead contact

Further information and requests for resources and materials should be directed to and will be fulfilled by the lead contact, Ib Chorkendorff (ibchork@fysik.dtu.dk).

#### Materials availability

The materials in this study will be made available upon reasonable request.

### Preparation of porous Cu electrode

The SS mesh (SS316, 325 × 2,300, McMASTER-CARR) was cut into 0.2–1 cm_geo_^2^ pieces and then spot welded with Cu wire (≥99.98%, Goodfellow) for electrical connection and used as WE. Two Pt mesh (1.5 cm_geo_^2^, 99.9%, Goodfellow) electrodes were connected and used as a split CE. The WE was located in the middle of the two Pt meshes. Prior to electrodeposition, the WE was dipped in 0.06 M HCl (VWR Chemicals) and rinsed with Milli-Q water and ethanol. The electrolyte was made of 0.4 M CuSO_4_ (98%, Merck) in 1.5 M H_2_SO_4_ (99.999%, Sigma Aldrich). A constant current of −2 A with applied time ranging from 15 s to 7 min was set for the porous Cu deposition on SS mesh. The electrode was cleaned with Milli-Q water and ethanol several times and dried under vacuum after electrodeposition. The excess deposited Cu on the Cu wire and the edge of SS mesh were removed to keep the constant geometric area of the WE at different experimental conditions (Figure S29). All the WEs were stored in an Ar glovebox before use.

### Electrochemical experiments

A three-electrode single-compartment glass cell in an electrochemical home-made autoclave placed in a fume hood was used for all the electrochemical experiments. The same electrochemical autoclave in an Ar glovebox was specifically used for the SEI investigation. Electrolyte solution (30 mL) was prepared in an Ar glovebox, which consists of 2 M lithium salts (99.99%, Sigma Aldrich) in 99 vol % THF (anhydrous, >99.9%, inhibitor-free, Sigma Aldrich) and 1 vol % ethanol (anhydrous, Honeywell). The glass cell, the magnetic stirring rotor (glass covered), CE (1.5 cm_geo_^2^ Pt mesh, 99.9%, Goodfellow), and RE (Pt wire, 99.99%, Goodfellow) were boiled in Milli-Q water and dried overnight at 100°C in air. The CE and RE are both flame-annealed before use, and the distance between WE and RE was fixed around 0.5 cm for all the experiments. Considering the convenience of running the experiment in the fume hood, which showed the same performance as those obtained from the autoclave inside the Ar glovebox, only the experiments associated with the investigation of the SEI layer were conducted in an autoclave placed inside an Ar glovebox to avoid air exposure. The Ar gas (99.999%, Air Liquide) was introduced into the assembled cell in the autoclave sitting in the fume hood for at least 30 min before the electrochemical experiment. Then, the electrolyte solution was injected into the cell in an Ar atmosphere, followed by the closing of the autoclave. Afterward, the pressure in the autoclave was increased to 10 bar using N_2_ (99.9999%, Air Liquide) and de-pressurized to 3 bar for 10 times in order to flush out any remaining air contaminants, followed by filling to 20 bar for the experiments. The N_2_ gas used here was cleaned by commercial purifiers (NuPure, pptV cleaning of all labile N containing compounds). The electrochemical measurements were started from an open circuit voltage (OCV) for 20 min to equilibrate the atmosphere composition in the electrolyte. Then, the PEIS, LSV, and CP, followed by another PEIS was applied for the measurements (Bio-Logic SP-200). The LSV was used to see a clear onset for lithium reduction, thereby denoting the potential versus Li/Li^+^ and confirming that the target current was reached before CP. During CP, varied constant-current densities from −0.075 to −1.0 A cm_geo_^−2^ were applied until the total passed charge reached 240 C. All the experiments were performed with the electrolyte stirred at 250 rpm at room temperature. The porous Cu electrodes with geometric surface area of 0.2 cm_geo_^2^ were used for all the electrochemical experiments considering the current and potential limit of the potentiostat. The porous Cu electrodes with 1.0 cm_geo_^2^ were used for the CP measurements at a current density of −0.1 A cm_geo_^−2^, which remains within the current and potential range of the potentiostat, and exhibit a FE similar to that of the electrodes with smaller geometric surface area.

### Separation procedure

All the separation procedures were conducted in an autoclave placed inside an Ar glovebox to ensure no exposure to air. Typically, after the electrochemical experiments, we took out the electrolyte through a sampling tube while the system was still pressurized at 20 bar, degassed the system through an acid trap (0.06 M HCl) to collect all the NH_3_ in the gas phase, and then removed the WE with the intact SEI layer in the end. To quantify the produced NH_3_ in the SEI layer, the WE was dipped into 10 mL of 0.06 M HCl to gradually dissolve the SEI layer.

### Colorimetric quantification of NH_3_

The produced NH_3_ was quantified by a modified colorimetric indophenol method.^11^ The calibration solutions consisted of known concentrations of NH_4_Cl in the dilute aqueous solution containing lithium salts (Figure S30). 500 μL of alkaline hypochlorite solution (A1727, Sigma Aldrich) was added to a 2 mL sample with NH_4_Cl, followed by the addition of 500 μL of phenol nitroprusside solution (P6994, Sigma Aldrich). The solution was then mixed and left in the dark for 30 min at room temperature, followed by the measurement of absorbance using UV-vis spectroscopy (UV-2600, Shimadzu) from 400 to 1,000 nm. The fitted calibration curve that shows a linear regression with an R^2^ value of 0.9999 was used for the quantifications (Figure S30). It is clearly shown that a high Li salt concentration (≥250 mM) has an obvious effect on the indophenol reaction. For example, a concentration of 0.5 M LiClO_4_ in the sample leads to a much lower slope in the calibration curve, which indicates that a falsely high FE might be calculated without proper dilution and calibration to eliminate the effect of the lithium salts concentration. Considering the produced NH_3_ at milligram level in this work, all the as-obtained samples after electrochemical experiments were diluted with Milli-Q water as needed to keep the measured absorbance located in the range of the calibration curve. For the sample from the electrolyte, 10 μL of 4 M HCl (37%, VWR Chemicals) was added to 500 μL of electrolyte and then diluted as required (ranging from 200 to 800 times). For the sample from the SEI and gas phase, the corresponding acid solutions were also diluted as needed (ranging from 10 to 200 times). All the FE were calculated by the following equation:

(Equation 1) FE = 3 × F × *c*_NH3_ × *V*/*Q*

where 3 is the number of electrons transferred for each mole of NH_3_, F is the Faraday constant, *c*_NH3_ is the concentration of produced NH_3_, *V* is the total electrolyte volume, and *Q* is the total passed charge.

To estimate the EE, we considered the total amount of energy put into the system via the potentiaostat, *E*_in_, and compared that with the energy contained in the total amount of NH_3_ produced during the experiment, *E*_out_. It should be noted that we do not include the pressure and energy from EtOH in our calculations. All the EE were calculated by the following equation:

(Equation 2) $$EE=\frac{E_{out}}{E_{in}}=\frac{\text{Δ}G_{r}\cdot m_{NH3}}{\int V_{cell}\left(t\right)\cdot I\left(t\right)dt}$$

where *E*_out_ was defined by the free energy (Δ*G*_r_) of reaction of NH_3_ oxidation to N_2_ and water times the amount of NH_3_ produced (*m*_NH3_), and *E*_in_ is given by the total cell voltage (*V*_cell_) between the CE and WE, multiplied by the current (*I*) to get the instantaneous power, and integrated over time.

### SEI investigation

XPS and XRD were used to investigate the SEI after electrochemistry. XPS was conducted on a ThermoScientific Thetaprobe instruments with an Al Kα X-ray source and base pressure below 9.0 × 10^−10^ mbar. The ion gun in etching mode and flood gun in charge neutralization mode were used during the measurement with a chamber pressure of 2.0 × 10^−7^ mbar by flowing Ar gas (99.9999%, Air Liquide). The ion gun was run using 4 kV and 1 μA mode with scanning size of 2 × 2 mm^2^. The spot size of 400 μm was used. All the survey spectra were recorded with step size of 1.0 eV and dwell time of 50 ms at pass energy of 200 eV (Figures S31 and S32). High-resolution elemental spectra were recorded with step size of 0.1 eV and dwell time of 50 ms at pass energy of 200 eV. All the spectra were acquired and analyzed by Thermo Avantage (v5.9925) by Thermo Fisher Scientific. All the background was determined using Shirley mode and fitted using Powell algorithm. To analyze all the powder samples with XPS, all the commercial powders were shaped to pellets with 7 mm diameter and 0.5–1 mm thickness in an Ar glovebox. The pellets were loaded in a custom-made sample holder in transfer arm. For the XRD measurements, the deposited layer was scraped off the electrode and loaded into the holder inside an Ar glovebox with an air-tight polyetheretherketone (PEEK) dome and then transferred to the XRD instrument without air exposure (Figure S33). XRD patterns were recorded on a Malvern Panalytical Empyrean X-ray diffractometer. The source was an Empyrean Cu LFF HR gun (Kα_1_ = 1.540598 Å) operated at 45 kV and 40 mA.

### Capacitive cycling experiment

The capacitive cycling experiments were conducted at ambient pressure using 2 M LiClO_4_ with the same electrode setup as the autoclave experiments. The CV measurements of different porous Cu electrodes and Cu foil were conducted at scan rates of 20–80 mV s^−1^. The specific capacitance (*C*_spec_, F cm^−2^) is the slope of the current density change (Δ*I*) versus scan rates shown in Figure 2F. The ΔI were calculated by the following equation:

(Equation 3) Δ*I* = (*I*_a_-*I*_c_)/2

where *I*_a_ and *I*_c_ is the anodic and cathodic current density at −0.5 V versus Pt pseudo-reference electrode, respectively.

### Theory calculations

Our calculations are based on density functional theory (DFT) within the generalized Kohn-Sham scheme,^49^ using the Vienna *ab initio* simulation package (VASP),^50^ as implemented in atomic simulation environment (ASE).^51^ We use the beef-vdw exchange-correlation functional^52^ to model adsorption properties as well as van der Waals interactions. We employ the Heyd-Scuseria-Ernzerhof (HSE06)^53^ with 25% mixing of short-range Hartree-Fock exchange to estimate the valence-band maximum (VBM) and conduction-band minimum (CBM). Projector augmented wave (PAW) potentials^54^^,^^55^ are used with a plane-wave cutoff of 600 eV. The smallest spacing between k points is chosen as 0.15 Å. The Li 1*s*^2^2*s*^1^, N 2*s*^2^2*p*^3^, O 2*s*^2^2*p*^4^, C 2*s*^2^2*p*^2^, F 2*s*^2^2*p*^5^, Cl 3*s*^2^3*p*^5^, and H 1*s*^2^ electrons are treated explicitly as valence. Calculations of migration barriers are based on the CI-NEB method.^30^

#### Defect calculations

To calculate defect properties, supercells are constructed with dimensions 2***a*** × 2***b*** × 2***c*** for Li_2_CO_3_, 3***a*** × 3***b*** × 2***c*** for LiOH, 3***a*** × 3***b*** × ***c*** for LiHF_2_, and 2***a*** × 2***b*** × 2***c*** for LiF. Different Li defects are investigated, including Li vacancies in different charge states and Li interstitials in different charge states. The *E*_f_ formation energy of a specific defect is calculated as follows:^56^

(Equation 4) *E*_f_ = *E*_q_ − *E*_bulk_ + *nμ*_Li_ + *qE*_F_ + Δ_corr_

Here, *E*_q_ represents the total energy of a supercell containing Li defect in charge state *q*, *E*_bulk_ is the total energy of a supercell containing no defect, *n* represents the number of Li atoms added (n < 0) or removed (n > 0) from the system, *μ* is the chemical potential of Li, *E*_F_ is the Fermi level that is a variable with values ranging from VBM to CBM, and Δ_corr_ is a finite-size correction factor^57^^,^^58^ neglected in this study.

#### Ionic conductivity

The ionic conductivity, σ, is calculated from the Nernst-Einstein equation:^29^

(Equation 5) $$\sigma =\frac{F^{2}q^{2}DS}{RT}=\frac{D_{0}e^{2}N_{sites}}{k_{B}T}\text{exp}\left(\frac{-\left(E_{f}+E_{m}\right)}{k_{B}T}\right)$$

Here, $D_{0}=\alpha \nu a^{2}\text{exp}\left(\frac{\text{Δ}S}{k_{B}}\right)$, where *α* is a geometry-related factor often close to unity, *ν* is the hopping frequency around a typical phonon frequency, *a* is the distance between sites, the entropy term Δ*S* is neglected in this study, and *E*_m_ is the migration barrier. The estimation error in Li conductivity is due to the general prediction error of the exchange-correlation functional.^59^

## Data Availability

The datasets generated in this study are available from the lead contact upon reasonable request.

## Supplemental information

Document S1. Figures S1–S33, Tables S1 and S2, and supplemental references

Document S2. Article plus supplemental information

## References

[1] United States Geological Survey (USGS) (2022). Mineral commodity summaries 2022: U.S.... nitrogen (fixed)–ammonia. https://pubs.usgs.gov/periodicals/mcs2022/mcs2022-nitrogen.pdf.

[2] MacFarlane D.R., Cherepanov P.V., Choi J., Suryanto B.H.R., Hodgetts R.Y., Bakker J.M., Ferrero Vallana F.M., Simonov A.N. (2020). A roadmap to the ammonia economy. Joule.

[3] Christensen C.H., Johannessen T., Sørensen R.Z., Nørskov J.K. (2006). Towards an ammonia-mediated hydrogen economy?. Catal. Today.

[4] Haber F., Le Rossignol R. (1913). Über die technische Darstellung von Ammoniak aus den Elementen. Z. Elektrochem. Angew. Phys. Chem..

[5] Erisman J.W., Sutton M.A., Galloway J., Klimont Z., Winiwarter W. (2008). How a century of ammonia synthesis changed the world. Nat. Geosci..

[6] van der Ham C.J.M., Koper M.T.M., Hetterscheid D.G.H. (2014). Challenges in reduction of dinitrogen by proton and electron transfer. Chem. Soc. Rev..

[7] Wismann S.T., Engbæk J.S., Vendelbo S.B., Bendixen F.B., Eriksen W.L., Aasberg-Petersen K., Frandsen C., Chorkendorff I., Mortensen P.M. (2019). Electrified methane reforming: A compact approach to greener industrial hydrogen production. Science.

[8] Fichter F., Girard P., Erlenmeyer H. (1930). Elektrolytische Bindung von komprimiertem Stickstoff bei gewöhnlicher Temperatur. Helv. Chim. Acta.

[9] Tsuneto A., Kudo A., Sakata T. (1993). Efficient electrochemical reduction of N_2_ to NH_3_ catalyzed by lithium. Chem. Lett..

[10] Tsuneto A., Kudo A., Sakata T. (1994). Lithium-mediated electrochemical reduction of high pressure N_2_ to NH_3_. J. Electroanal. Chem..

[11] Andersen S.Z., Čolić V., Yang S., Schwalbe J.A., Nielander A.C., McEnaney J.M., Enemark-Rasmussen K., Baker J.G., Singh A.R., Rohr B.A. (2019). A rigorous electrochemical ammonia synthesis protocol with quantitative isotope measurements. Nature.

[12] Choi J., Suryanto B.H.R., Wang D., Du H.-L., Hodgetts R.Y., Ferrero Vallana F.M., MacFarlane D.R., Simonov A.N. (2020). Identification and elimination of false positives in electrochemical nitrogen reduction studies. Nat. Commun..

[13] Andersen S.Z., Statt M.J., Bukas V.J., Shapel S.G., Pedersen J.B., Krempl K., Saccoccio M., Chakraborty D., Kibsgaard J., Vesborg P.C.K. (2020). Increasing stability, efficiency, and fundamental understanding of lithium-mediated electrochemical nitrogen reduction. Energy Environ. Sci..

[14] Lazouski N., Schiffer Z.J., Williams K., Manthiram K. (2019). Understanding continuous lithium-mediated electrochemical nitrogen reduction. Joule.

[15] Lazouski N., Chung M., Williams K., Gala M.L., Manthiram K. (2020). Non-aqueous gas diffusion electrodes for rapid ammonia synthesis from nitrogen and water-splitting-derived hydrogen. Nat. Catal..

[16] Schwalbe J.A., Statt M.J., Chosy C., Singh A.R., Rohr B.A., Nielander A.C., Andersen S.Z., McEnaney J.M., Baker J.G., Jaramillo T.F. (2020). A combined theory-experiment analysis of the surface species in lithium-mediated NH_3_ electrosynthesis. ChemElectroChem.

[17] Cherepanov P.V., Krebsz M., Hodgetts R.Y., Simonov A.N., MacFarlane D.R. (2021). Understanding the factors determining the faradaic efficiency and rate of the lithium redox-mediated N_2_ reduction to ammonia. J. Phys. Chem. C.

[18] Suryanto B.H.R., Matuszek K., Choi J., Hodgetts R.Y., Du H.L., Bakker J.M., Kang C.S.M., Cherepanov P.V., Simonov A.N., MacFarlane D.R. (2021). Nitrogen reduction to ammonia at high efficiency and rates based on a phosphonium proton shuttle. Science.

[19] Iriawan H., Andersen S.Z., Zhang X., Comer B.M., Barrio J., Chen P., Medford A.J., Stephens I.E.L., Chorkendorff I., Shao-Horn Y. (2021). Methods for nitrogen activation by reduction and oxidation. Nat. Rev. Methods Primers.

[20] Cai X., Fu C., Iriawan H., Yang F., Wu A., Luo L., Shen S., Wei G., Shao-Horn Y., Zhang J. (2021). Lithium-mediated electrochemical nitrogen reduction: mechanistic insights to enhance performance. iScience.

[21] Li K., Andersen S.Z., Statt M.J., Saccoccio M., Bukas V.J., Krempl K., Sažinas R., Pedersen J.B., Shadravan V., Zhou Y. (2021). Enhancement of lithium-mediated ammonia synthesis by addition of oxygen. Science.

[22] Dey A.N. (1977). Lithium anode film and organic and inorganic electrolyte batteries. Thin Solid Films.

[23] Peled E. (1979). The electrochemical behavior of alkali and alkaline earth metals in nonaqueous battery systems-the solid electrolyte interphase model. J. Electrochem. Soc..

[24] Li K., Shapel S.G., Hochfilzer D., Pedersen J.B., Krempl K., Andersen S.Z., Sažinas R., Saccoccio M., Li S., Chakraborty D. (2022). Increasing current density of Li-mediated ammonia synthesis with high surface area copper electrodes. ACS Energy Lett..

[25] Wagman D.D., Evans W.H., Parker V.B., Schumm R.H., Halow I. (1982). The NBS tables of chemical thermodynamic properties. Selected values for inorganic and C1 and C2 organic substances in SI units. J. Phys. Chem. Ref. Data.

[26] Piller F.M., Appukkuttan P., Gavryushin A., Helm M., Knochel P. (2008). Convenient preparation of polyfunctional aryl magnesium reagents by a direct magnesium insertion in the presence of LiCl. Angew. Chem. Int. Ed. Engl..

[27] Shi S., Lu P., Liu Z., Qi Y., Hector L.G., Li H., Harris S.J. (2012). Direct calculation of Li-ion transport in the solid electrolyte interphase. J. Am. Chem. Soc..

[28] Ramesh S., Bing K.N. (2012). Conductivity, mechanical and thermal studies on poly(methyl methacrylate)-based polymer electrolytes complexed with lithium tetraborate and propylene carbonate. J. Mater. Eng. Perform..

[29] Maier J. (2004).

[30] Henkelman G., Uberuaga B.P., Jónsson H. (2000). A climbing image nudged elastic band method for finding saddle points and minimum energy paths. J. Chem. Phys..

[31] Winter M. (2009). The solid electrolyte interphase-the most important and the least understood solid electrolyte in rechargeable Li batteries. Z. Phys. Chem..

[32] Wang E., Dey S., Liu T., Menkin S., Grey C.P. (2020). Effects of atmospheric gases on Li metal cyclability and solid-electrolyte interphase formation. ACS Energy Lett..

[33] Lu Y., Tu Z., Archer L.A. (2014). Stable lithium electrodeposition in liquid and nanoporous solid electrolytes. Nat. Mater..

[34] Dey A.N. (1971). Electrochemical alloying of lithium in organic electrolytes. J. Electrochem. Soc..

[35] Herraiz-Cardona I., Ortega E., Vázquez-Gómez L., Pérez-Herranz V. (2012). Double-template fabrication of three-dimensional porous nickel electrodes for hydrogen evolution reaction. Int. J. Hydrog. Energy.

[36] Wang J., Shao H., Ren S., Hu A., Li M. (2021). Fabrication of porous Ni-Co catalytic electrode with high performance in hydrogen evolution reaction. Appl. Surf. Sci..

[37] Zhuo K., Jeong M.-G., Chung C.-H. (2013). Highly porous dendritic Ni-Sn anodes for lithium-ion batteries. J. Power Sources.

[38] Plowman B.J., Jones L.A., Bhargava S.K. (2015). Building with bubbles: the formation of high surface area honeycomb-like films via hydrogen bubble templated electrodeposition. Chem. Commun. (Camb).

[39] Sažinas R., Andersen S.Z., Li K., Saccoccio M., Krempl K., Pedersen J.B., Kibsgaard J., Vesborg P.C.K., Chakraborty D., Chorkendorff I. (2021). Towards understanding of electrolyte degradation in lithium-mediated non-aqueous electrochemical ammonia synthesis with gas chromatography-mass spectrometry. RSC Adv.

[40] Campion C.L., Li W., Lucht B.L. (2005). Thermal decomposition of LiPF_6_-based electrolytes for lithium-ion batteries. J. Electrochem. Soc..

[41] Herstedt M., Abraham D.P., Kerr J.B., Edström K. (2004). X-ray photoelectron spectroscopy of negative electrodes from high-power lithium-ion cells showing various levels of power fade. Electrochim. Acta.

[42] Lee H.H., Wan C.C., Wang Y.Y. (2004). Thermal stability of the solid electrolyte interface on carbon electrodes of lithium batteries. J. Electrochem. Soc..

[43] Hong E.-S., Okada S., Sonoda T., Gopukumar S., Yamaki J.-i. (2004). Thermal stability of electrolytes with mixtures of LiPF_6_ and LiBF_4_ used in lithium-ion cells. J. Electrochem. Soc..

[44] Smith C., Hill A.K., Torrente-Murciano L. (2020). Current and future role of Haber-Bosch ammonia in a carbon-free energy landscape. Energy Environ. Sci..

[45] Hennessy J., Nikzad S. (2018). Atomic layer deposition of lithium fluoride optical coatings for the ultraviolet. Inorganics.

[46] Lou P., Li C., Cui Z., Guo X. (2016). Job-sharing cathode design for Li-O_2_ batteries with high energy efficiency enabled by in situ ionic liquid bonding to cover carbon surface defects. J. Mater. Chem. A.

[47] Mohtasebi A., Chowdhury T., Hsu L.H.H., Biesinger M.C., Kruse P. (2016). Interfacial charge transfer between phenyl-capped aniline tetramer films and iron oxide surfaces. J. Phys. Chem. C.

[48] Wagner C.D., Naumkin A.V., Kraut-Vass A., Allison J.W., Powell C.J., Rumble J.R. (2003). NIST Standard Reference Database 20, version 3.4 (web version)..

[49] Kohn W., Sham L.J. (1965). Self-consistent equations including exchange and correlation effects. Phys. Rev..

[50] Kresse G., Furthmüller J. (1996). Efficient iterative schemes for ab initio total-energy calculations using a plane-wave basis set. Phys. Rev. B Condens. Matter.

[51] Bahn S.R., Jacobsen K.W. (2002). An object-oriented scripting interface to a legacy electronic structure code. Comput. Sci. Eng..

[52] Wellendorff J., Lundgaard K.T., Møgelhøj A., Petzold V., Landis D.D., Nørskov J.K., Bligaard T., Jacobsen K.W. (2012). Density functionals for surface science: exchange-correlation model development with Bayesian error estimation. Phys. Rev. B.

[53] Heyd J., Scuseria G.E., Ernzerhof M. (2003). Hybrid functionals based on a screened Coulomb potential. J. Chem. Phys..

[54] Blöchl P.E. (1994). Projector augmented-wave method. Phys. Rev. B Condens. Matter.

[55] Kresse G., Joubert D. (1999). From ultrasoft pseudopotentials to the projector augmented-wave method. Phys. Rev. B.

[56] Freysoldt C., Grabowski B., Hickel T., Neugebauer J., Kresse G., Janotti A., Van de Walle C.G. (2014). First-principles calculations for point defects in solids. Rev. Mod. Phys..

[57] Freysoldt C., Neugebauer J., Van de Walle C.G. (2009). Fully ab initio Finite-size corrections for charged-defect supercell calculations. Phys. Rev. Lett..

[58] Freysoldt C., Neugebauer J., Van de Walle C.G. (2011). Electrostatic interactions between charged defects in supercells. Phys. Status Solidi B..

[59] Medford A.J., Wellendorff J., Vojvodic A., Studt F., Abild-Pedersen F., Jacobsen K.W., Bligaard T., Nørskov J.K. (2014). Catalysis. Assessing the reliability of calculated catalytic ammonia synthesis rates. Science.

