# Supplementary material for: Electrosynthesis of ammonia with high selectivity and high rates via engineering of the solid-electrolyte interphase
Source: Joule. 2022 Sep 21;6(9):2083–101. doi: 10.1016/j.joule.2022.07.009 (PMC9511958; doi:10.1016/j.joule.2022.07.009)
Supplement: Document S1. Figures S1–S33, Tables S1 and S2, and supplemental references [file mmc1.pdf]

Joule, Volume *6*

Supplemental information

Electrosynthesis of ammonia with high selectivity

and high rates via engineering

# of the solid-electrolyte interphase

Shaofeng Li, Yuanyuan Zhou, Katja Li, Mattia Saccoccio, Rokas Sažinas, Suzanne Z. Andersen, Jakob B. Pedersen, Xianbiao Fu, Vahid Shadravan, Debasish Chakraborty, Jakob Kibsgaard, Peter C.K. Vesborg, Jens K. Nørskov, and Ib Chorkendorff

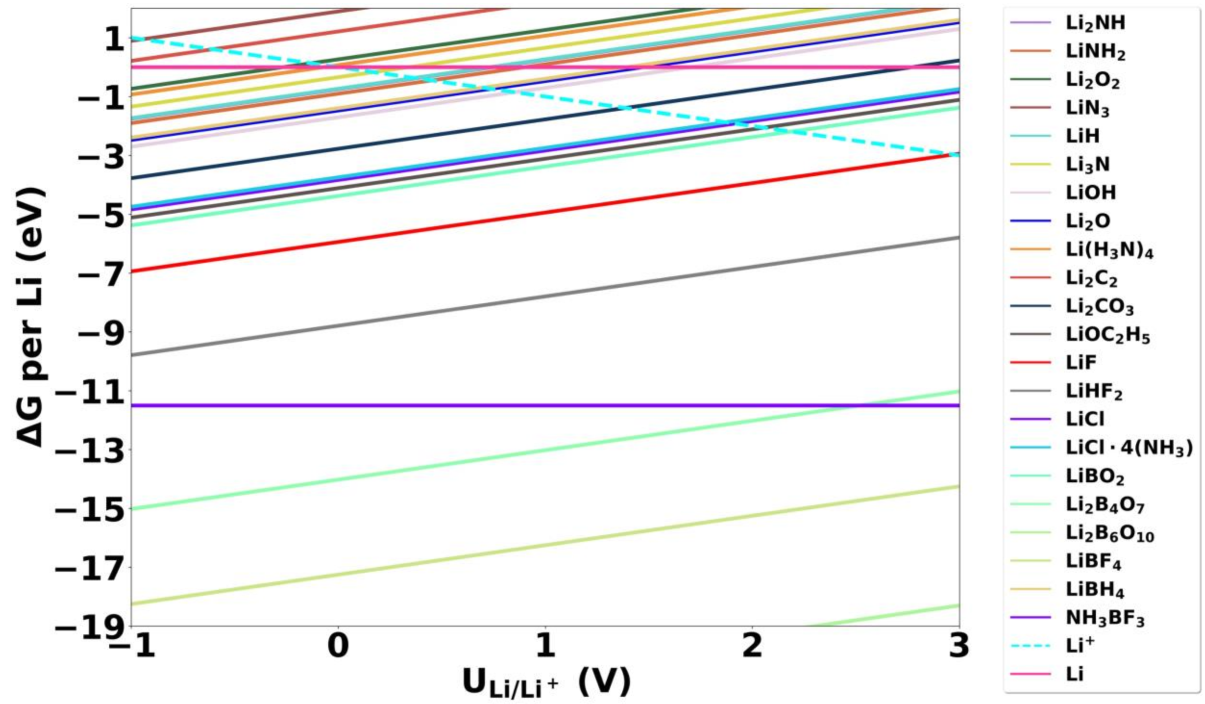

Figure S1. Calculated Gibbs formation free energy of Li-containing compounds as a function of voltage (vs Li/Li^+). The partial pressure of NH_3, H_2O, C_2H_5OH, Cl_2 and F_2 was set to 0.1, 10^{-7}, 10^{-5}, 10^{-5}, 10^{-5} bar, respectively.

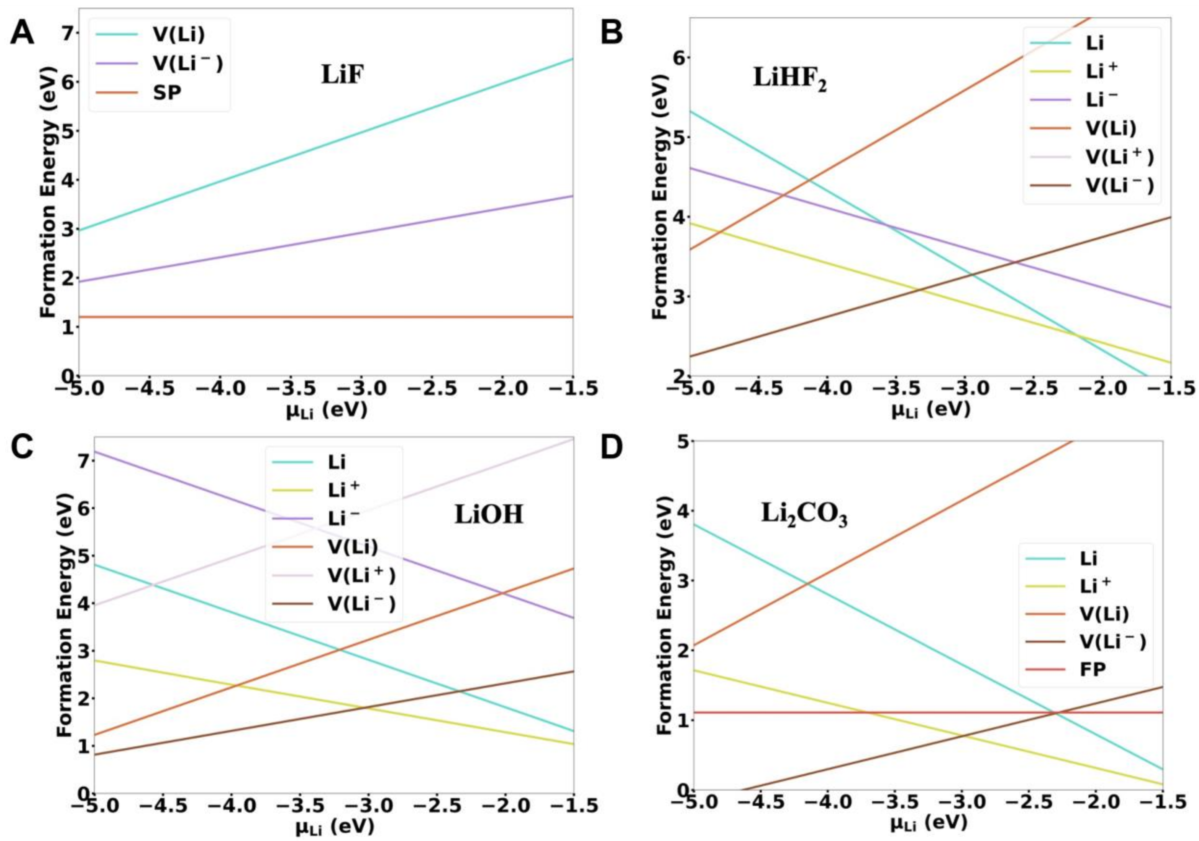

Figure S2. Formation energies for different point defects in LiF (A), $LiHF_2$ (B), LiOH (C) and Li_2CO_3 (D). V(Li), V(Li^+) and V(Li^-): Li vacancies in 0, +1 and -1 charge states, respectively. Li, Li^+ and Li^-: Li interstitials in 0, +1 and -1 charge states. SP: neutral Schottky pair (V(Li)+V(F)). FP: Li neutral Frenkel pair.

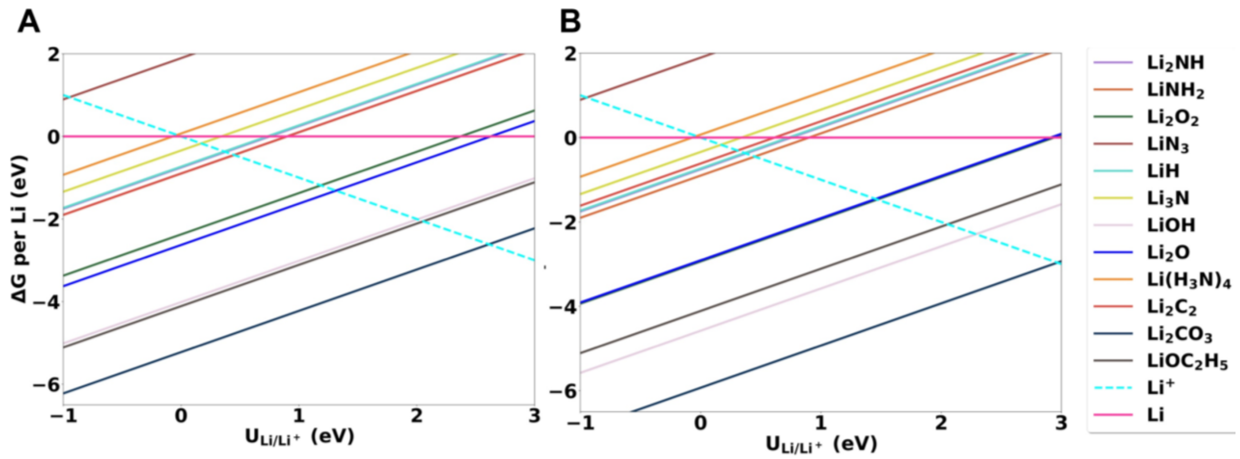

Figure S3. Calculated Gibbs formation free energy of Li-containing compounds as a function of voltage (vs Li/Li^+) at O_2 partial pressure of 10^{-10} (A) and 0.1 bar (B). The partial pressure of NH_3, O_2, C_2H_5OH was set to 0.1, 1, 10^{-5} bar, respectively.

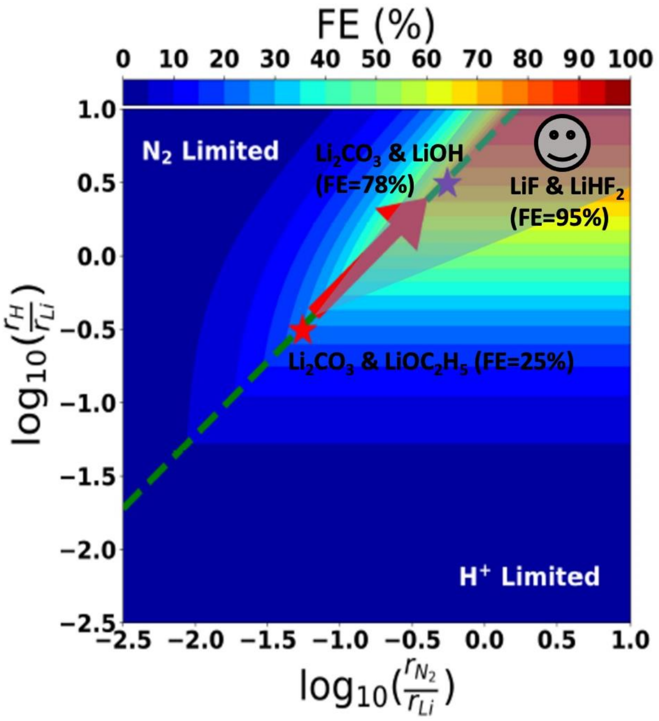

Figure S4. Heatmap of the predicted FE as a function of the ratio of nitrogen to lithium (*x* axis) and proton to lithium (*y* axis) diffusion rates. The red star indicates the expected location of the 10-bar experiments without O_2 in the system in Ref[1]. The purple star indicates the improvement in FE if r_{Li} were selectively lowered by an order of magnitude when the LiOH (10^{-16} S cm^{-1}) becomes competitive in the SEI. The emoji indicates the substantial increase in FE if r_{Li} were significantly lowered by an order of magnitude when the LiF (10^{-30} S cm^{-1}) and $LiHF_2$ (8.47×10^{-24}) become the two main components in the SEI. The cone represents the uncertainty of the location of the purple star and the emoji. The increase in FE is based on the assumption that there is a relatively small change of r_H and r_{N2} (**Table S1**) compared to r_{Li}.

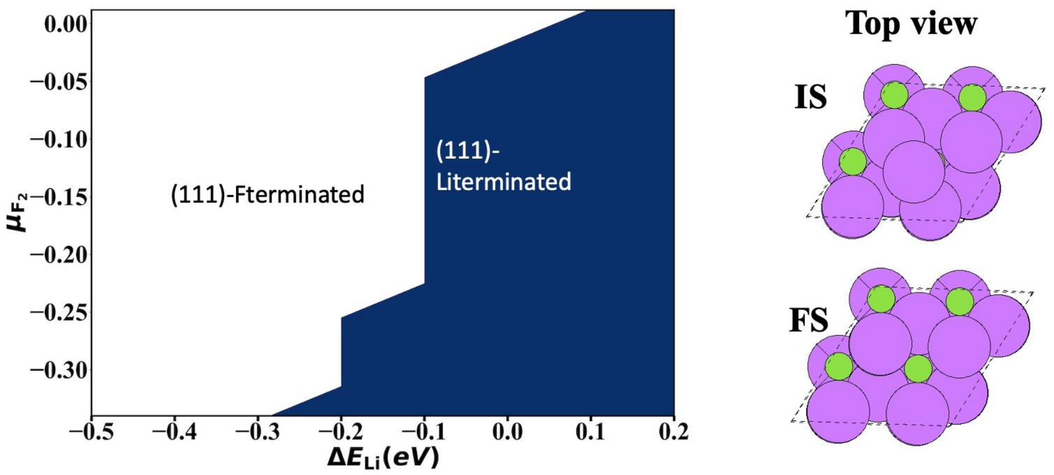

Figure S5. Left panel: surface phase diagram of LiF as the function of Li and F_2 chemical potentials. Right panel: initial state (IS) and final state (FS) of the top view of Li-terminated LiF(111) for Li diffuses on the surface.

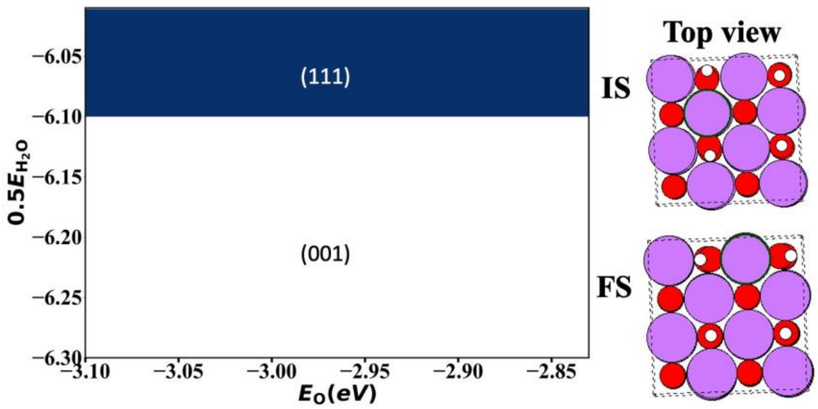

Figure S6. Left panel: surface phase diagram of LiOH with respect to bulk Li_2O, O_2 and H_2O. Right panel: initial state (IS) and final state (FS) of the top view of LiOH(111) for Li diffuses on the surface.

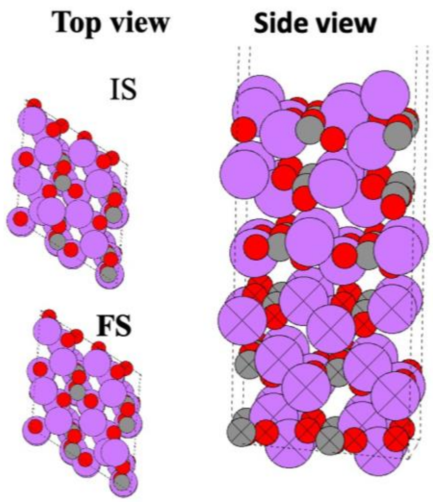

Figure S7. Left panel: initial state (IS) and final state (FS) of the top view of Li_2CO_3(001) surface for Li diffuses on the surface. Right panel: the side view of Li_2CO_3(001) surface.

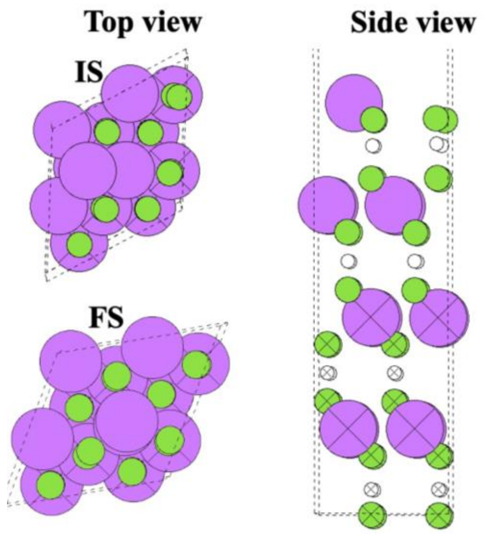

Figure S8. Left panel: initial state (IS) and final state (FS) of the top view of $LiHF_2$(001) surface for Li diffuses on the surface. Right panel: the side view of $LiHF_2$(001) surface.

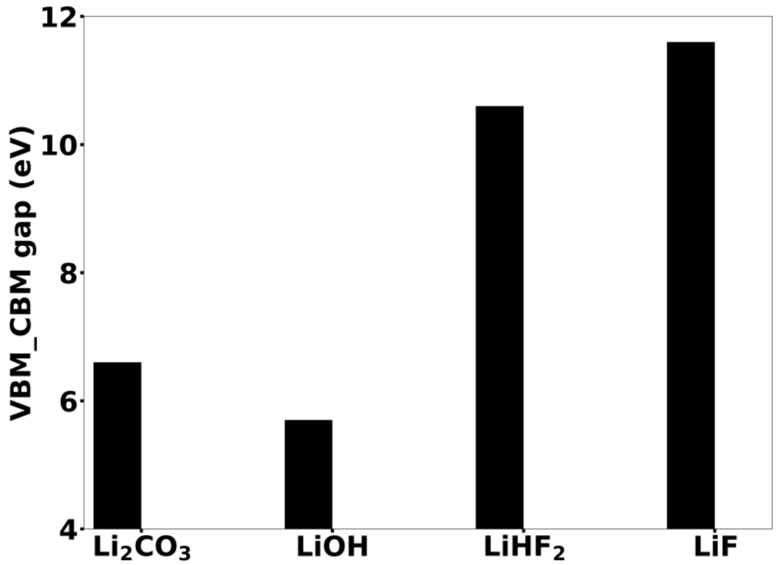

Figure S9. The gap between valence-band maximum (VBM) conduction-band minimum (CBM) estimated based on the Heyd-Scuseria-Ernzerhof (HSE06),[2] with 25% mixing of short-range Hartree-Fock exchange approximation for Li_2CO_3, LiOH, $LiHF_2$ and LiF.

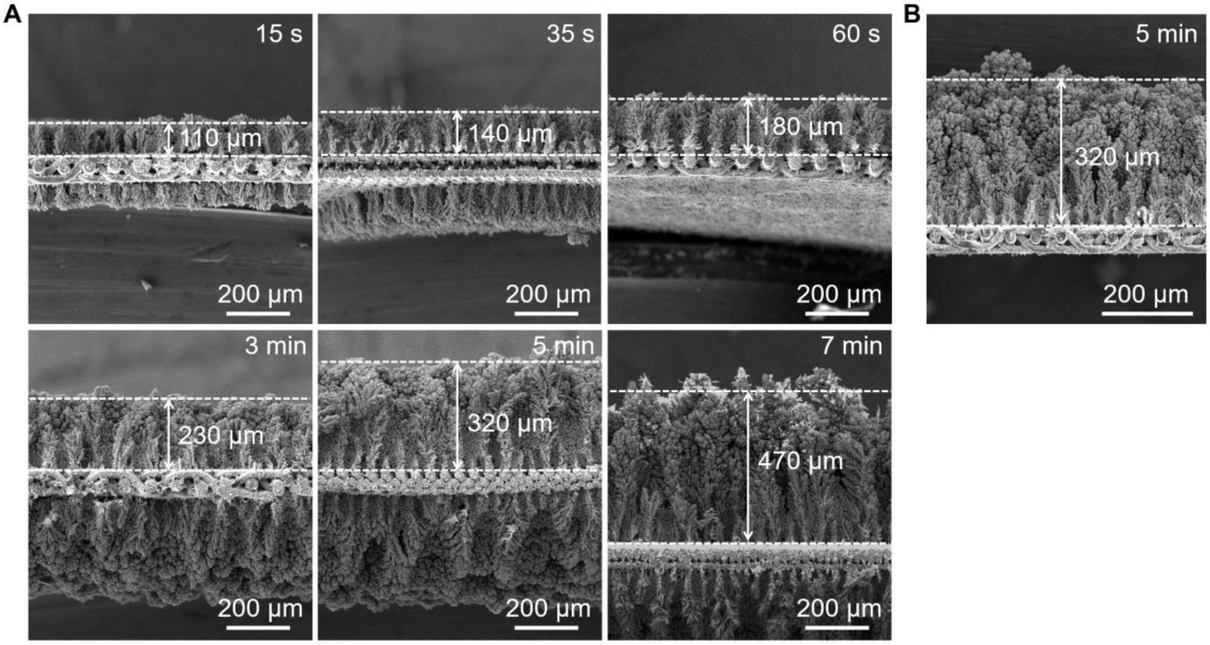

Figure S10. A, B, SEM images of the porous Cu electrode synthesized at varied deposition time ranging from 15 s to 7 min (A) and the porous Cu electrode synthesized at deposition time of 5 min with backside deposited Cu removed (B).

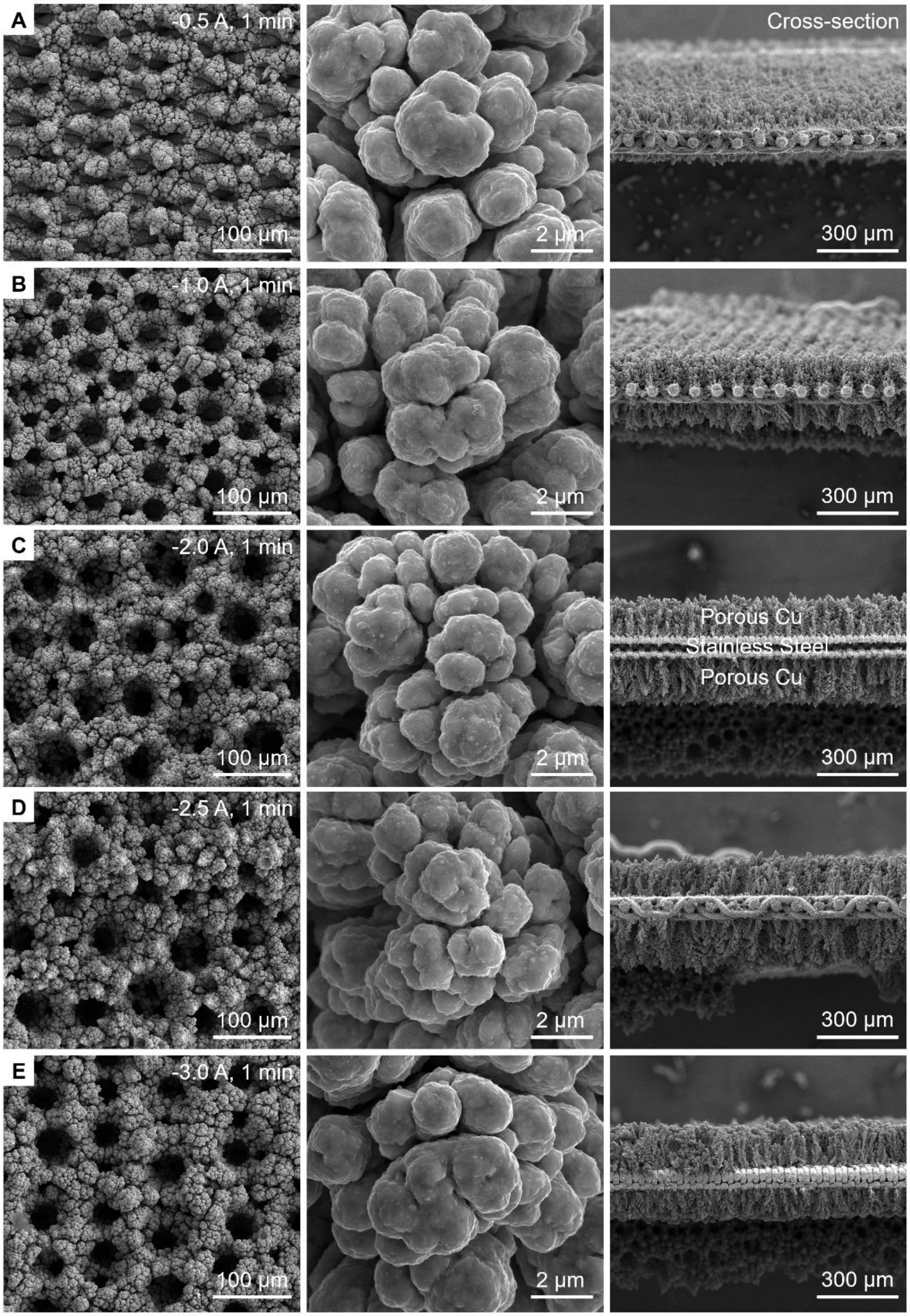

Figure S11. SEM images of the porous Cu electrode synthesized at varied applied current ranging from -0.5 to -3.0 A.

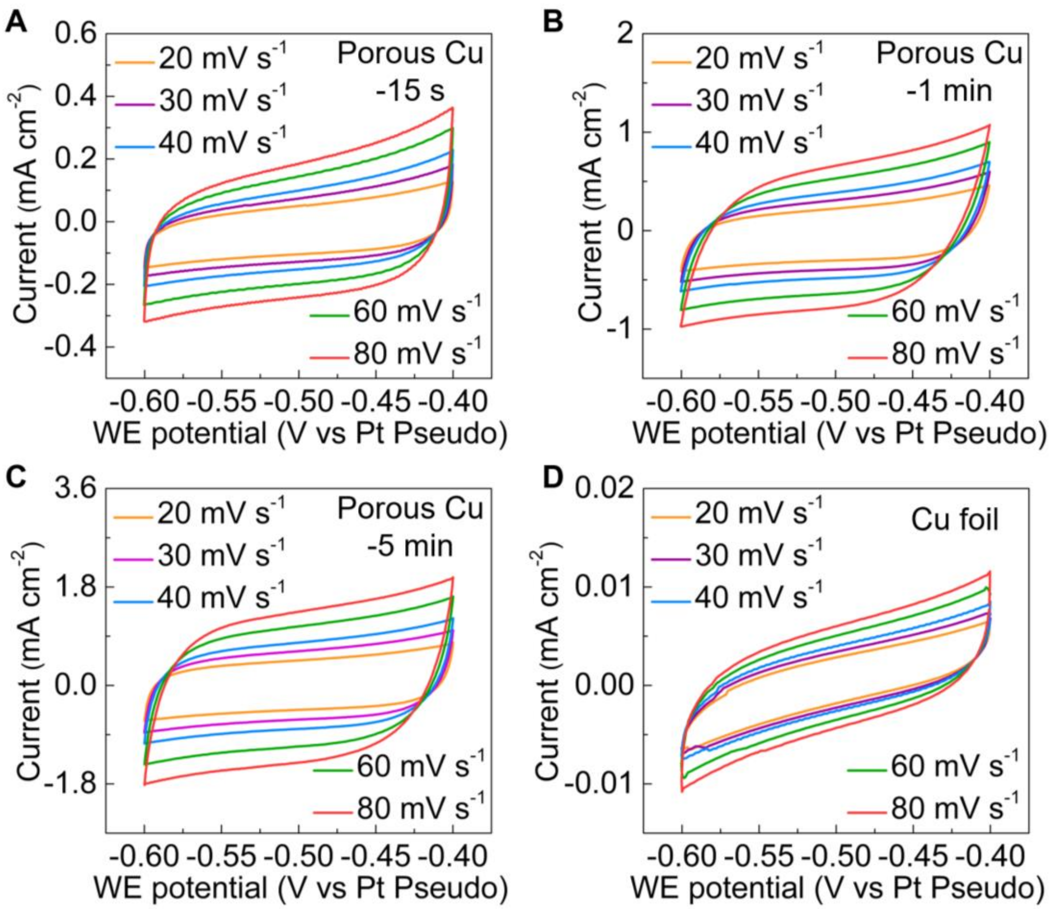

Figure S12. Cyclic voltammetry of different Cu electrodes at various scan rates ranging from 20 to 80 mV s^{-1}.

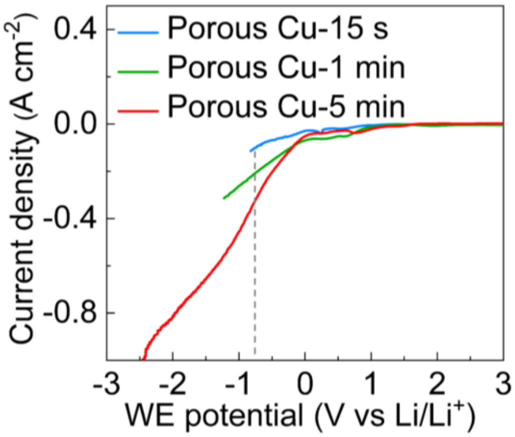

Figure S13. LSV curves of the porous Cu electrodes with deposition time of 15 s, 1 min and 5 min. The $LiClO_4$-based electrolyte were used here to investigate the current density achievable.

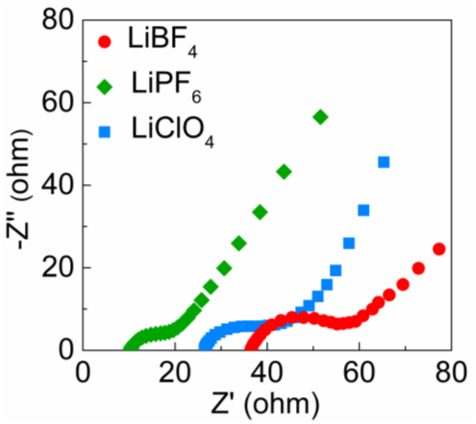

Figure S14. Nyquist plots of the porous Cu electrode using different lithium salts.

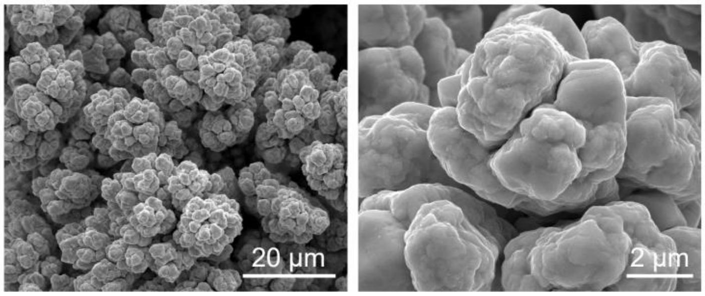

Figure S15. SEM images of the porous Cu electrodes using $LiBF_4$ after CP measurement at a current density of -1.0 A cm_{geo}^{-2}.

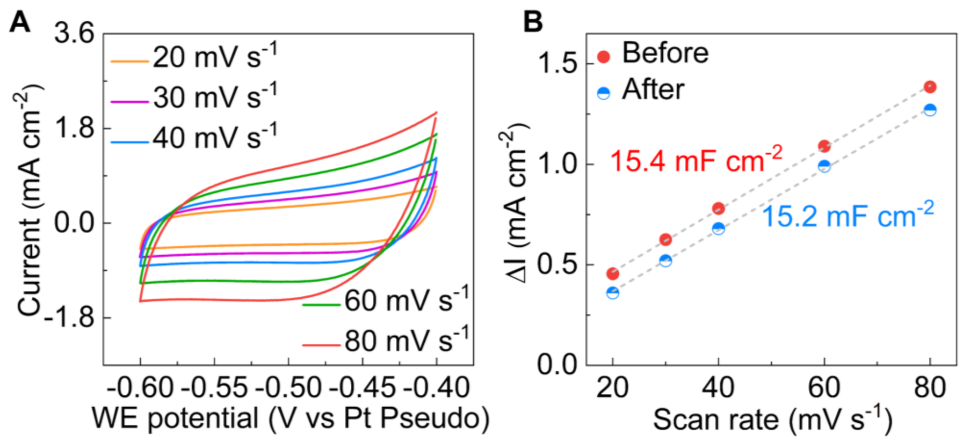

Figure S16. Cyclic voltammetry (A) and current density change versus scan rate (B) of the porous Cu electrodes using $LiBF_4$ after CP measurement at a current density of -1.0 A cm_{geo}^{-2}.

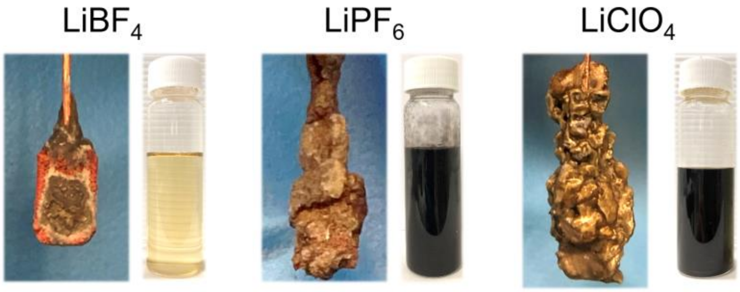

Figure S17. Digital photos of the porous Cu electrode with deposit (left) and electrolyte (right) after CP measurement when using $LiBF_4$, $LiPF_6$ and $LiClO_4$ at current densities of -1.0 A cm_{geo}^{-2}.

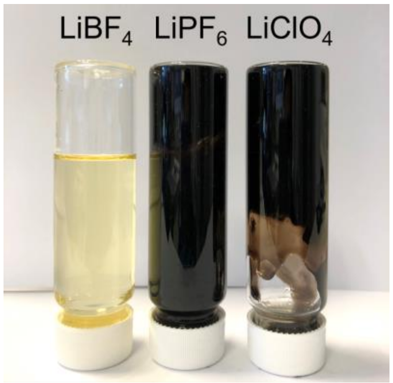

Figure S18. Digital photos of the electrolytes a few hours after CP measurement when using $LiBF_4$, $LiPF_6$ and $LiClO_4$ at current densities of -1.0 A cm_{geo}^{-2}. The electrolytes visibly changed color, and in the cases of both $LiPF_6$ and $LiClO_4$, became highly viscous.

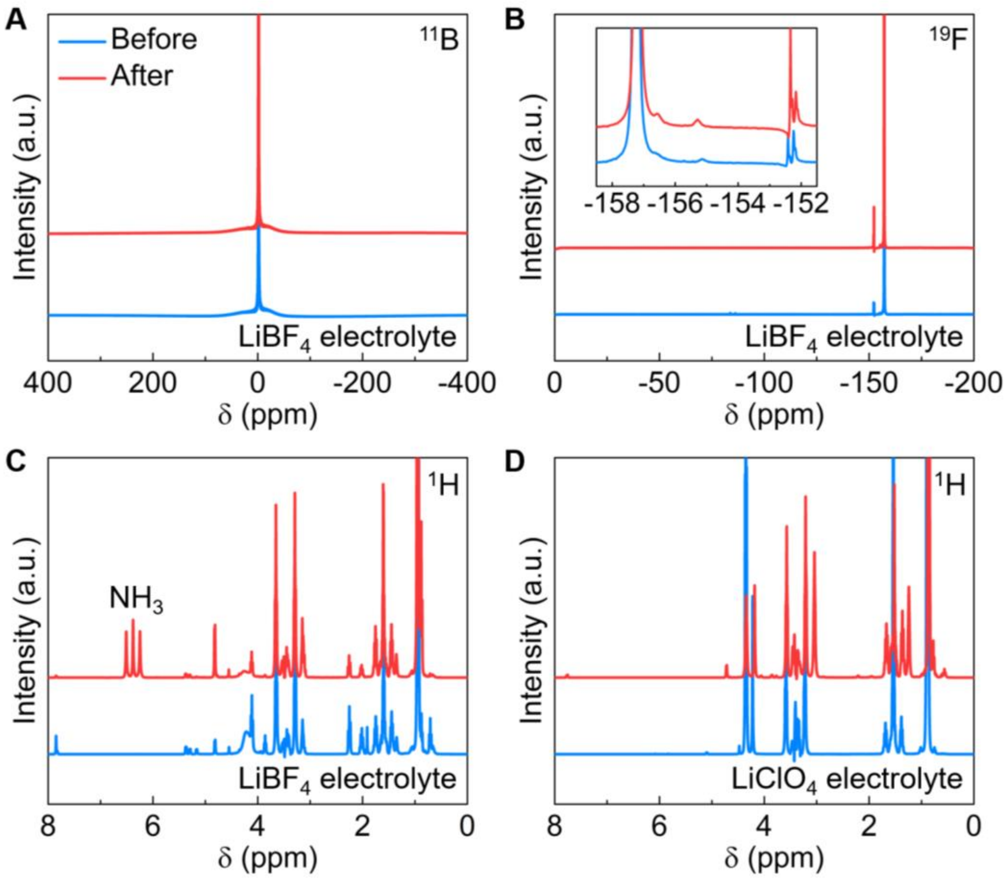

Figure S19. A-C, ^{11}B (A), ^{19}F (B), and ^1H (C) NMR spectra of electrolyte using $LiBF_4$ salt before and after CP measurement at -1.0 A cm_{geo}^{-2}. D, ^1H NMR spectra of electrolyte using $LiClO_4$ salt before and after CP measurement at -1.0 A cm_{geo}^{-2}. The curve name in (B-D) is identical to (A). It is clearly to see that the no new peaks are shown in the ^{11}B, ^{19}F, and ^1H NMR spectra of $LiBF_4$ electrolyte after CP measurement, except the NH_3 signal shown in ^1H NMR spectra. However, more new peaks appeared in the ^1H NMR spectra of $LiClO_4$ electrolyte after CP measurement, which indicates severe electrolyte decomposition.

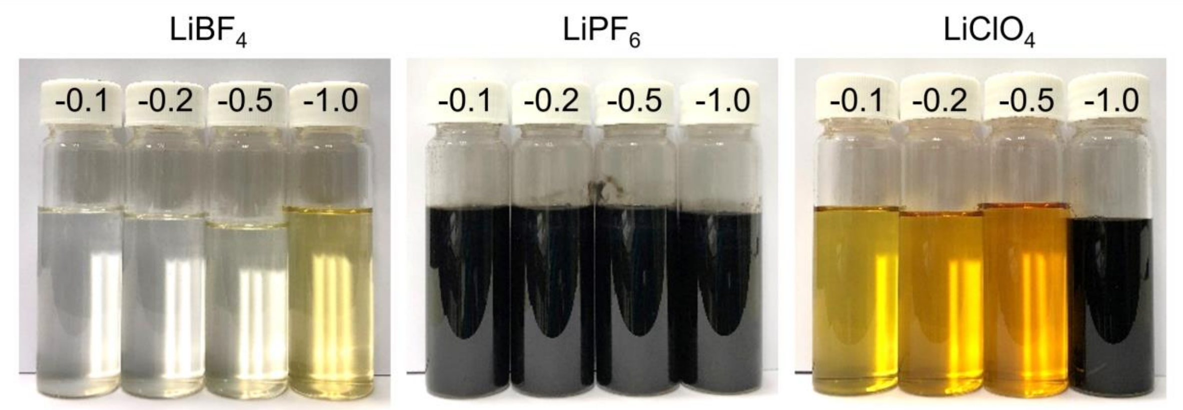

Figure S20. Digital photos of the electrolyte after CP measurement by using $LiBF_4$, $LiPF_6$ and $LiClO_4$ at varied current densities from -0.1 to -1.0 A cm_{geo}^{-2}.

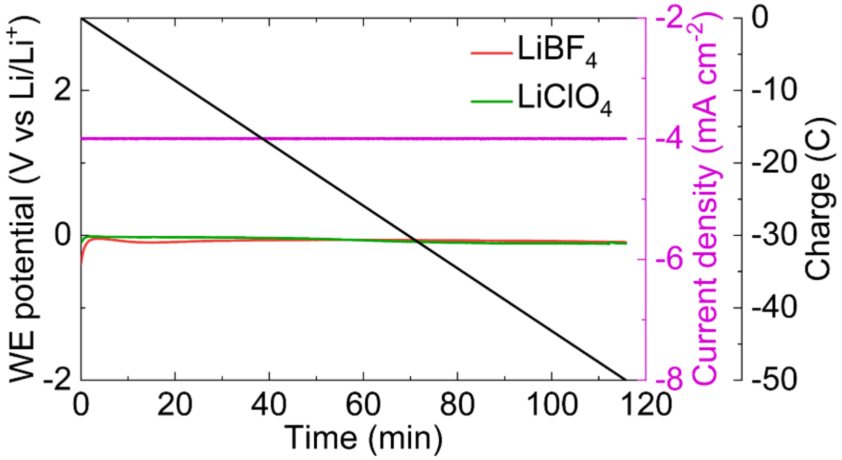

Figure S21. CP of the Cu foil at current density of -4 mA cm^{-2} with different lithium salts.

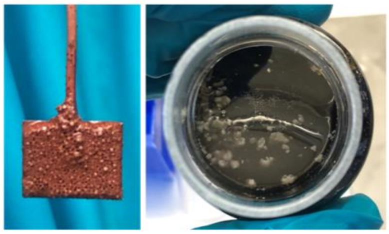

Figure S22. Digital photos of the porous Cu electrode (left) and electrolyte (right) after depressurization from 20 bar without separation procedure after CP measurement when using $LiClO_4$ at current density of -1.0 A cm_{geo}^{-2}.

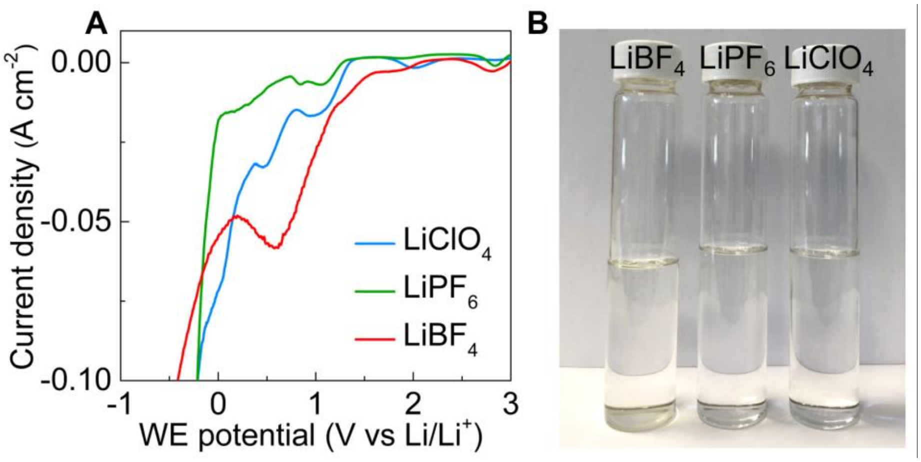

Figure S23. A, LSV curves of the porous Cu electrodes using $LiBF_4$, $LiPF_6$ and $LiClO_4$ salts. B, Digital photos of the different electrolytes after LSV measurements shown in (A).

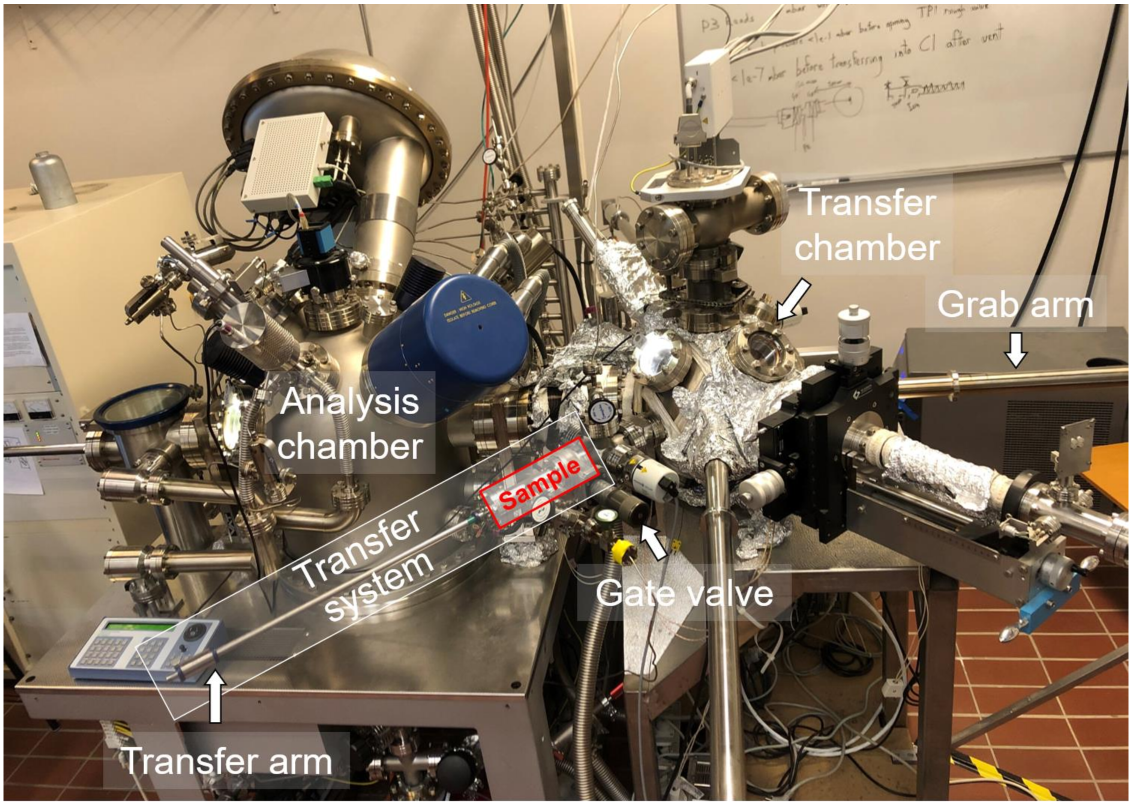

Figure S24. Digital photo of the home-built XPS transfer system. The transfer system was first loaded into an Ar glovebox for sample loading, and the gate valve on the system was close. Then the system was attached to the transfer chamber and pumped down. When the pressure of the transfer system has reached below 5×10^{-6} mbar, the transfer gate is opened and sample was introduced to the transfer chamber. Finally, the grab arm is used to catch the sample and transfer it to the analysis chamber.

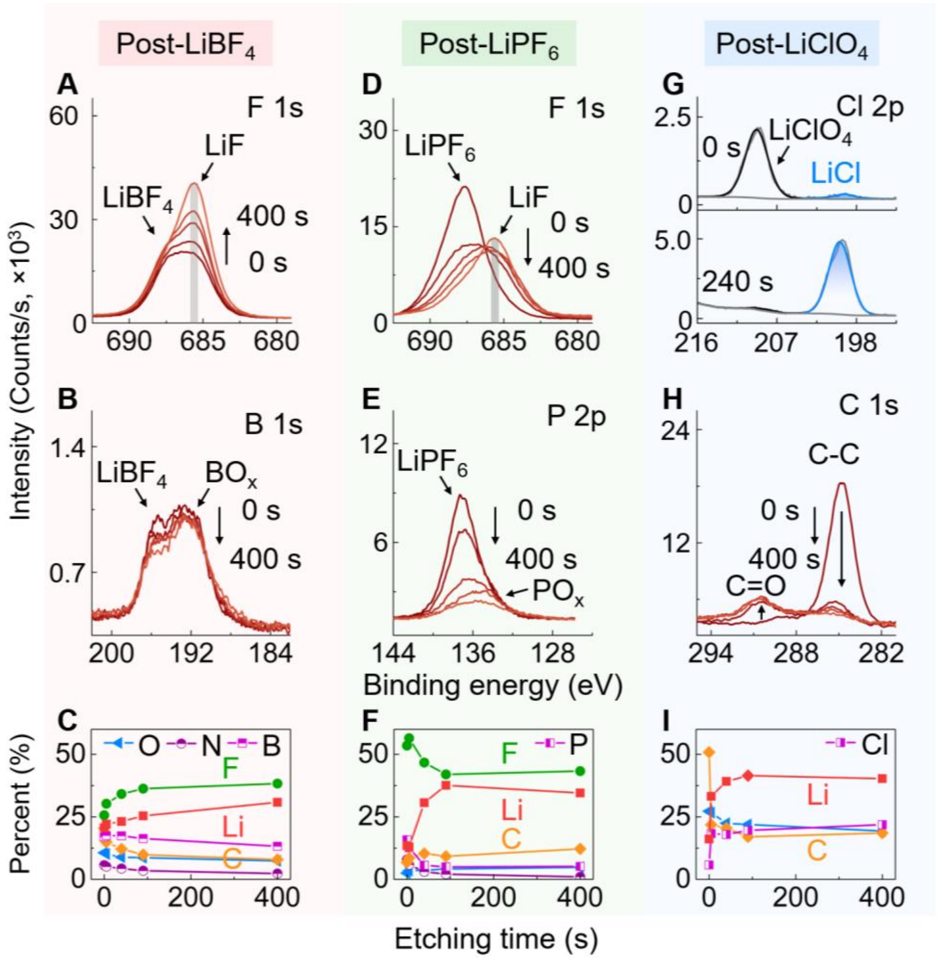

Figure S25. XPS investigation on the deposit after electrochemistry at -1.0 A cm_{geo}^{-2}. A-C, Depth-profiling XPS spectra of F 1s (A), B 1s (B) and elemental composition (C) for the Post-$LiBF_4$. D-F, Depth-profiling XPS spectra of F 1s (D), P 2p (E) and elemental composition (F) for the Post-$LiPF_6$. G-I, Depth-profiling XPS spectra of Cl 2p (G), C 1s (H) and elemental composition (I) for the Post-$LiClO_4$.

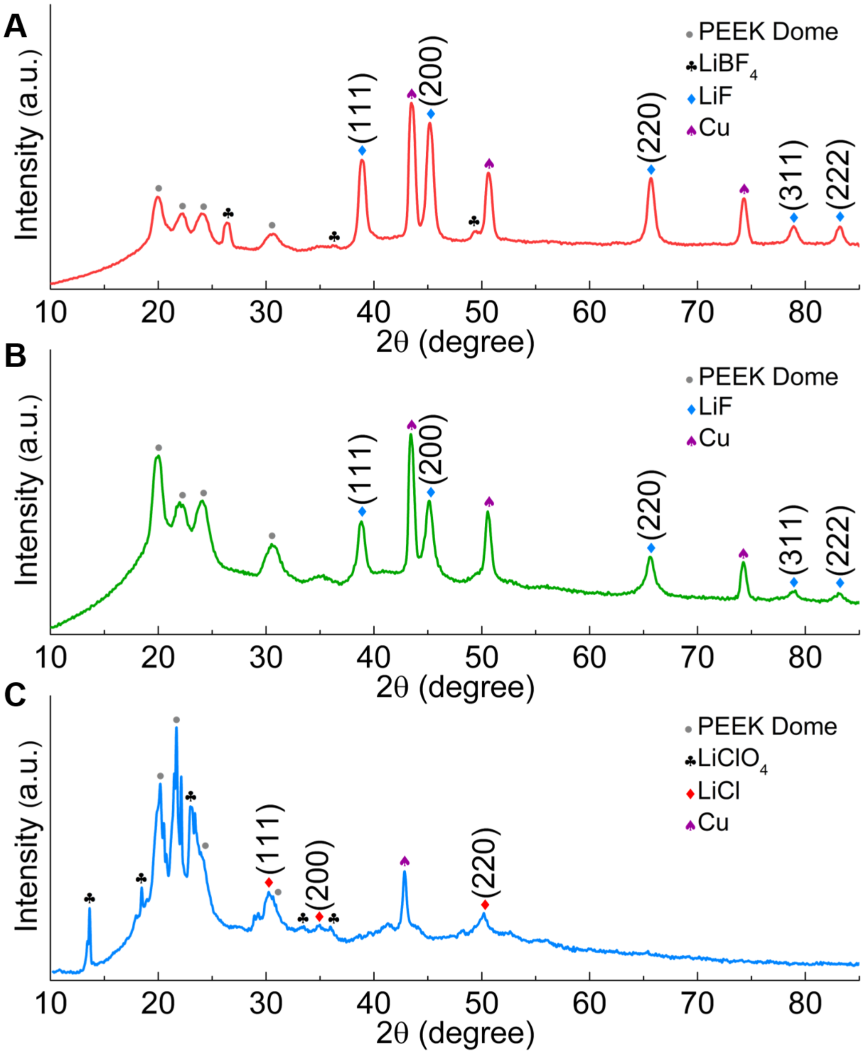

Figure S26. A-C, XRD patterns of Post-$LiBF_4$ (A), Post-$LiPF_6$ (B), and Post-$LiClO_4$ (C). The LiF (ICSD: 98-005-3839) is clearly shown in the Post-$LiBF_4$ and Post-$LiPF_6$.

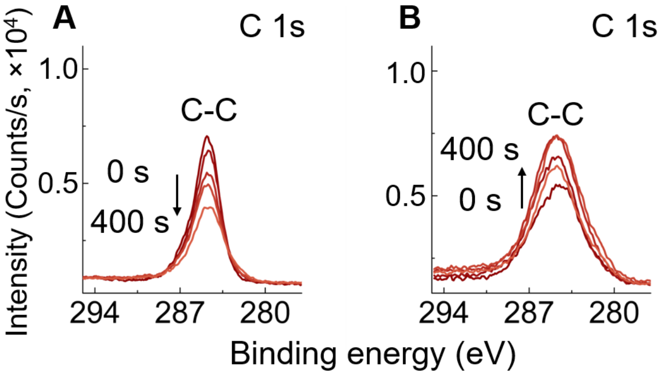

Figure S27. A, B, Depth-profiling XPS spectra of C 1s for the Post-$LiBF_4$ (A) and Post-$LiPF_6$ (B).

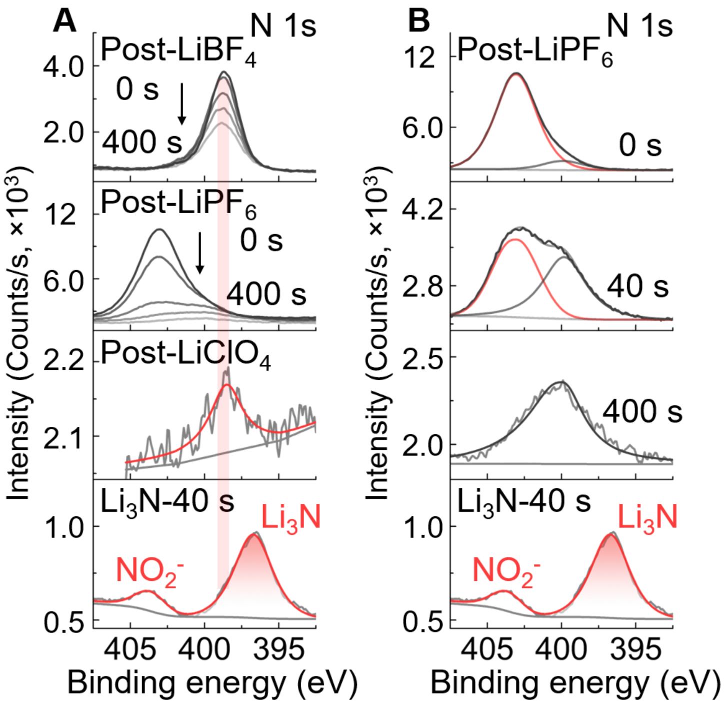

Figure S28. Depth-profiling XPS spectra of N 1s for the deposit formed using different lithium salts (A) and $LiPF_6$ (B) after CP measurements at a current density of -1.0 A cm_{geo}^{-2}. The commercial Li_3N powder were used as reference samples. The weak N 1s signal of the SEI-$LiClO_4$ may be caused by a low concentration of the nitrogen species in the thicker SEI layer with more organic compounds, which could decompose or volatilize under ultrahigh vacuum conditions (such as those inside the XPS chamber). It is also noted that the SEI-$LiPF_6$ shows a peak attributed to the nitrite species on the surface, and the N 1s signal does not only decreases rapidly during etching but also shows new peak centered at 400.0 eV. The nitrite species may be caused by NH_3 oxidation during the reaction, which will be further investigated in a future study.

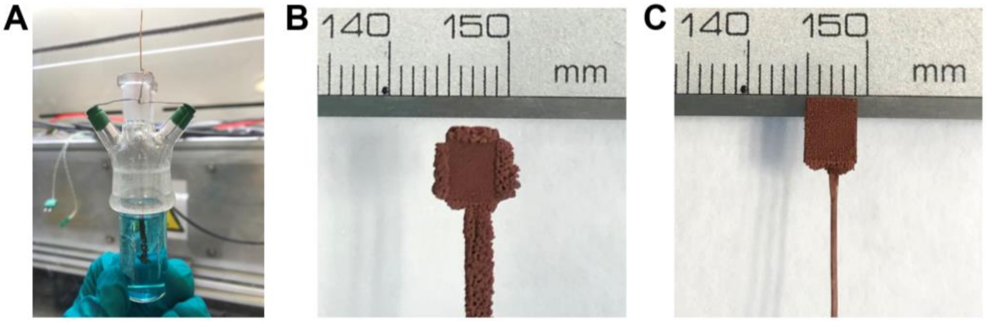

Figure S29. A, Digital photos of the cell with electrodes after Cu electrodeposition. B, C, Digital photos of the porous Cu electrode before (B) and after (C) removing the excess Cu deposited on the Cu wire and the edge of SS mesh.

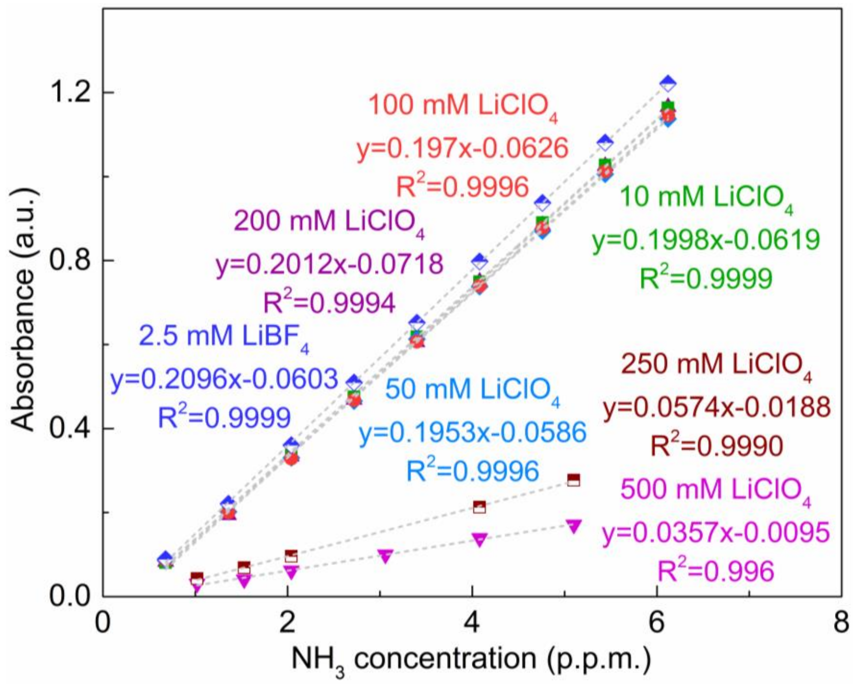

Figure S30. Calibration curves of known concentrations of NH_4Cl in dilute aqueous solution containing lithium salts. The fitted calibration curve using dilute aqueous solution containing 2.5 mM $LiBF_4$ that shows a linear regression with an R^2 value of 0.9999 was used for the quantifications. It should be noted that higher Li salt concentration (≥250 mM) has an obvious effect on the indophenol reactions, which should be avoided for the colorimetric indophenol method.

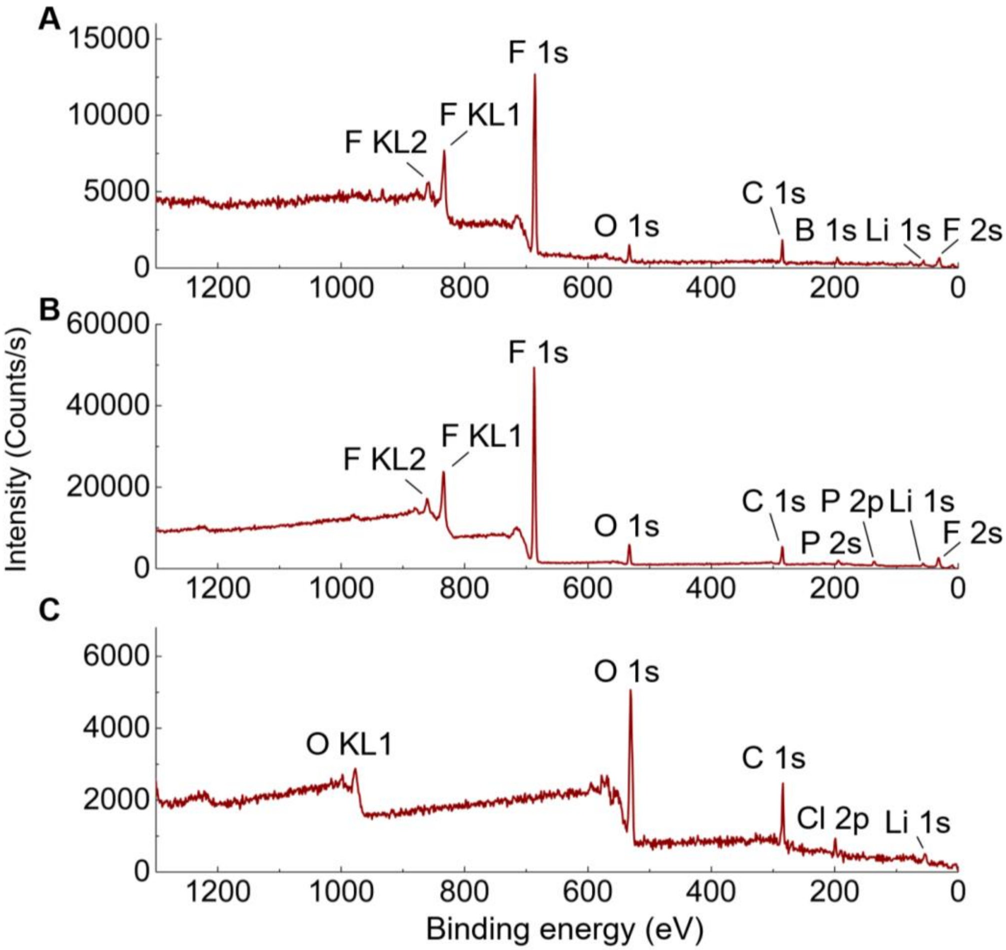

Figure S31. A-C, XPS survey spectra of SEI-$LiBF_4$ (A), SEI-$LiPF_6$ (B), and SEI-$LiClO_4$ (C).

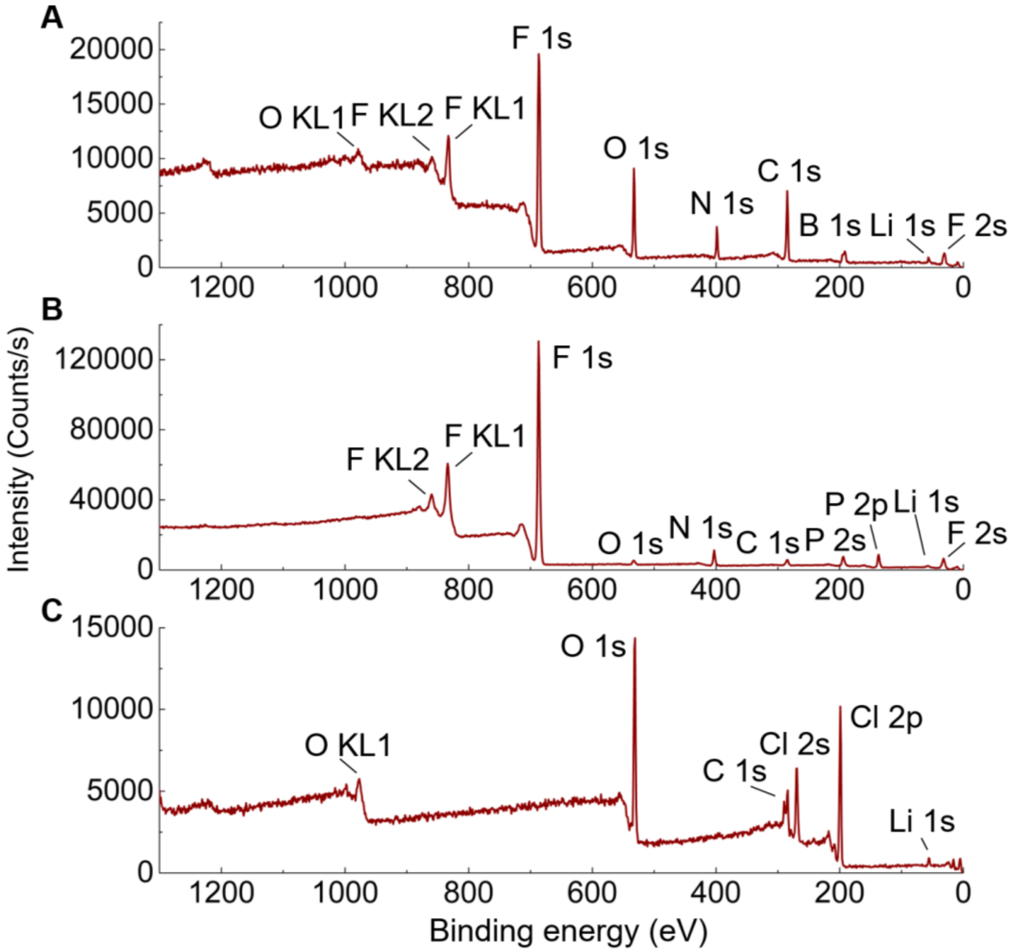

Figure S32. A-C, XPS survey spectra of Post-$LiBF_4$ (A), Post-$LiPF_6$ (B), and Post-$LiClO_4$ (C).

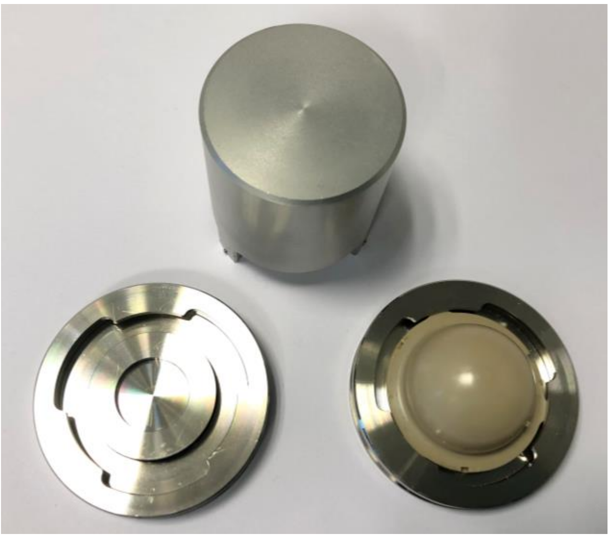

Figure S33. Digital photos of the XRD sample holder with PEEK dome used for XRD measurement without air exposure. The thin and X-ray transparent PEEK dome (Anton Paar, Cat. No. 132601, X-ray transparency 70%) is tightened onto the holder base (Anton Paar, Cat. No. 132598), and the air-tightness is ensured by an O-ring between the dome and sample holder. The photo also shows the mounting tool necessary to fix the dome onto the base.

Table S1. The diffusion rates of proton and N_2 are estimated via Fick's first law. Consider the case of linear (one-dimension) diffusion of proton from bulk electrolyte through SEI approaching the electrode surface, the flux of proton $J_{H^+}(x,t)$ at given position x at a time t is proportional to the concentration gradient C_{H^+}, that is, $J_{H^+}(x,t) = D_{H^+}\frac{\partial C_{H^+}(x,t)}{\partial x}$. The thickness L of SEI is chosen to be 10-100 nm.[3] Since the nitrogen reduction and hydrogen evolution reactions are fast enough at the very negative potential (<-3 V), the proton and N_2 concentration at electrode surface ($x = L$) is approximated to be zero. Therefore, the diffusion rates of proton and nitrogen are estimated by $D_{H^+} = \frac{3J_{NH_3}}{FE}\frac{L}{C_{H^+}(0,t)}$ and $D_{N_2} = J_{NH_3}\frac{L}{C_{H^+}(0,t)}$.

	J_{NH_3}(mol cm^{-2} s^{-1})	D_{H^+} (cm^2 s^{-1})	D_{N_2}(cm^2 s^{-1})
Ref[1] -without O_2	$(3.3\pm0.05)\times10^{-9}$	2.3×10^{-9}-2.3×10^{-8}	1.9×10^{-10}-1.9×10^{-9}
Ref[1] -with O_2	$(10.8\pm0.05)\times10^{-9}$	2.6×10^{-10}-2.6×10^{-9}	0.66×10^{-10}-0.66×10^{-9}
This work	$(3.3\pm0.01)\times10^{-7}$	5.8×10^{-9}-5.8×10^{-8}	2.0×10^{-9}-2.0×10^{-8}

The ammonia production rate (J_{NH_3}) at -0.1 A cm_{geo}^{-2} was used here for this work.

Table S2. The EE of the systems under different conditions.

Samples	FE (%)	Cell voltage (V)	EE (%)
$LiBF_4$, -1.0 A cm^{-2}_{geo}	71±3	10.9	7.7±0.3
$LiPF_6$, -1.0 A cm^{-2}_{geo}	45±3	6.3	8.4±0.6
$LiClO_4$, -1.0 A cm^{-2}_{geo}	31±3	9.6	3.8±0.4

References

1. Li, K., Andersen Suzanne, Z., Statt Michael, J., Saccoccio, M., Bukas Vanessa, J., Krempl, K., Sažinas, R., Pedersen Jakob, B., Shadravan, V., Zhou, Y., Chakraborty, D., Kibsgaard, J., Vesborg Peter, C.K., Nørskov Jens, K., and Chorkendorff, I. (2021). Enhancement of lithium-mediated ammonia synthesis by addition of oxygen. Science *374*, 1593-1597.
2. Heyd, J., Scuseria, G.E., and Ernzerhof, M. (2003). Hybrid functionals based on a screened Coulomb potential. J. Chem. Phys. *118*, 8207-8215.
3. Winter, M. (2009). The solid electrolyte interphase-the most important and the least understood solid electrolyte in rechargeable Li batteries. Z. Phys. Chem. *223*, 1395-1406.
